# The prefoldin complex stabilizes the von Hippel-Lindau protein against aggregation and degradation

**DOI:** 10.1371/journal.pgen.1009183

**Published:** 2020-11-02

**Authors:** Franck Chesnel, Anne Couturier, Adrien Alusse, Jean-Philippe Gagné, Guy G. Poirier, Dominique Jean, François-Michel Boisvert, Pauline Hascoet, Luc Paillard, Yannick Arlot-Bonnemains, Xavier Le Goff

**Affiliations:** 1 Univ Rennes, CNRS, IGDR (Institut de génétique et développement de Rennes)—UMR 6290, France; 2 Department of Molecular Biology, Medical Biochemistry and Pathology; Université Laval, Québec City, Québec, Canada; 3 CHU de Québec Research Center, CHUL Pavilion, Oncology Axis, Québec City, Québec, Canada; 4 Department of Anatomy and Cell Biology, Université de Sherbrooke, Sherbrooke, Québec, Canada; University Medical Center Göttingen, ISRAEL

## Abstract

Loss of von Hippel-Lindau protein pVHL function promotes VHL diseases, including sporadic and inherited clear cell Renal Cell Carcinoma (ccRCC). Mechanisms controlling pVHL function and regulation, including folding and stability, remain elusive. Here, we have identified the conserved cochaperone prefoldin complex in a screen for pVHL interactors. The prefoldin complex delivers non-native proteins to the chaperonin T-complex-protein-1-ring (TRiC) or Cytosolic Chaperonin containing TCP-1 (CCT) to assist folding of newly synthesized polypeptides. The pVHL-prefoldin interaction was confirmed in human cells and prefoldin knock-down reduced pVHL expression levels. Furthermore, when pVHL was expressed in *Schizosaccharomyces pombe*, all prefoldin mutants promoted its aggregation. We mapped the interaction of prefoldin with pVHL at the exon2-exon3 junction encoded region. Low levels of the PFDN3 prefoldin subunit were associated with poor survival in ccRCC patients harboring VHL mutations. Our results link the prefoldin complex with pVHL folding and this may impact VHL diseases progression.

## Author summary

The von Hippel Lindau (VHL) tumor suppressor gene, when mutated, is responsible for the VHL disease, a genetic syndrome predisposing to cancer, and plays a critical early role in the development of sporadic kidney cancers. In this work, we show that the pVHL protein, the product of the VHL gene, interacts with the prefoldin complex. The function of this complex is to allow the correct folding of newly synthesized proteins. We show that, in the absence of prefoldin, pVHL is prone to aggregate or to be degraded because of its misfolding. Furthermore, the expression levels of prefoldin subunit PFDN3 are critical in kidney cancer patients harboring VHL mutations. Thus, our work identifies an important new player for the pVHL protein to fulfill its functions in the cell.

## Introduction

Mutations in the *von Hippel Lindau* (VHL) gene are responsible for the VHL disease, an autosomal dominant cancer syndrome that predisposes to various benign and malignant tumors, including clear cell Renal Cell Carcinoma (ccRCC), hemangioblastoma or pheochromocytoma [1,2]. Importantly, the VHL gene is also mutated or deleted in 80% of sporadic ccRCC, the most common subtype of kidney cancer [3]. The human VHL gene encodes three different protein isoforms from two transcripts. The mRNA variant 1 encodes the full-length pVHL213 and a shorter pVHL160 version produced from an internal translation start site. The mRNA variant 2 encodes pVHL172 in which exon 2 is skipped by the splicing machinery. The pVHL213 and pVHL160 isoforms have tumor suppressor activity unlike the pVHL172 isoform [4–6]. The pVHL213 and pVHL160 isoforms are essential substrate recognition subunits of an E3 ligase complex called VBC which also contains Elongins B and C [7,8]. Among the VBC complex substrates, the HIF alpha hypoxia-inducible transcription factor (HIFα) is considered as the main target. HIFα controls transcription of many genes including proliferation and angiogenesis stimulating genes and VHL inactivation promotes HIFα constitutive signaling. However pVHL also exerts a number of HIF-independent functions [9]. About one third of all VHL mutations are missense mutations producing full-length protein with altered functionality [10]. Although mutations in pVHL interfering with either HIFα and ElonginBC interactions have been characterized, many missense VHL mutations lie outside of known functional pVHL domains raising the possibility that some of these mutations influence pVHL folding and stability by promoting misfolded pVHL degradation [11,12].

By protecting hydrophobic solvent-exposed patches on the surface of unfolded proteins, chaperones facilitate folding and prevent aggregation of polypeptides in the crowded environment of the cytoplasm. Misfolded proteins are detected by protein quality control pathways that target them to the protein degradation machinery to prevent the formation of cytotoxic aggregates. A major task of this quality control system is performed by the prefoldin complex which takes the form of a jellyfish-like hexameric cochaperone conserved from archae to eukaryotes [13]. Eukaryotic hetero hexameric prefoldin complex consists of two alpha (PFDN3, PFDN5) and four beta subunits (PFDN1, PFDN2, PFDN4 and PFDN6). PFD subunits are composed of N- and C-terminal α-helical coiled-coil structures linked by either one (β subunits) or two (α subunits) β hairpins (reviewed in [14]). The prefoldin complex delivers non-native proteins to the group II chaperonin T-complex-protein-1-ring (TRiC) or Cytosolic Chaperonin containing TCP-1 (CCT) to assist folding of newly synthesized polypeptides such as actin and tubulin and to prevent their aggregation [15–20].

In budding yeast, the GIM1-GIM6 genes encoding the prefoldin subunits have been shown to regulate tubulin and actin folding [21–24]. In fission yeast, only two subunit functions has been deciphered yet. Pfd5, also known as Bob1, regulates the sexual differentiation signaling through the Byr1 MAPK [25]. We recently showed that Pfd3 also known as Pac10 controls heterogeneously expressed human pVHL expression levels [26]. Other putative prefoldin encoding genes *pfd1*, *pfd2*, *pfd4*, *pfd6* are yet to be characterized factors in fission yeast.

Individual folding-independent functions have also been attributed to some prefoldin subunits like c-Myc transcription for PFDN5 [27] or cyclin A expression for PFDN1 [28]. The PFDN3 subunit was initially called VBP1 for “von Hippel-Lindau Binding Protein 1” when it was identified as a new binding partner of pVHL carboxy-terminal region [29]. Binding of PFDN3 stabilizes pVHL and thus facilitates HIF1α degradation [30]. Another study of the *Drosophila merry-go-round mgr* mutant, corresponding to the PFDN3 homologue gene, suggested a cooperation between fruit fly VHL and PFDN3 homologues in tubulin biogenesis and folding [31]. However, whether these activities of PFDN3 towards pVHL are mediated through the folding function of the whole prefoldin complex was not established.

In this work, we show that pVHL associates with all six prefoldin subunits in a BioID screen. Using an expression system in fission yeast where prefoldin chaperone function is conserved, we show that the whole prefoldin complex protects pVHL213 from aggregation. Expression of the full-length human PFDN3 subunit, but not a chaperone-deficient mutant, restores pVHL solubility in a yeast *pfd3*Δ mutant. In human cells, down-regulation of the whole prefoldin complex through PFDN3 silencing reduces wild-type pVHL expression levels. We have identified that the region encoded by *VHL* at the junction of exon2 and exon3 allows the major binding of the prefoldin complex. In ccRCC TCGA database, a low PFDN3 expression level is correlated with poor survival in patients harboring missense mutated VHL. Our data suggest that prefoldin interaction with pVHL is a key step in the folding process of pVHL to reach its native conformation. Thus, prefoldin function may have an impact on pVHL proteostasis and this should influence VHL-related disease progression.

## Results

### The prefoldin complex associates preferentially with the pVHL213 isoform in human cells

**Fig 1:**
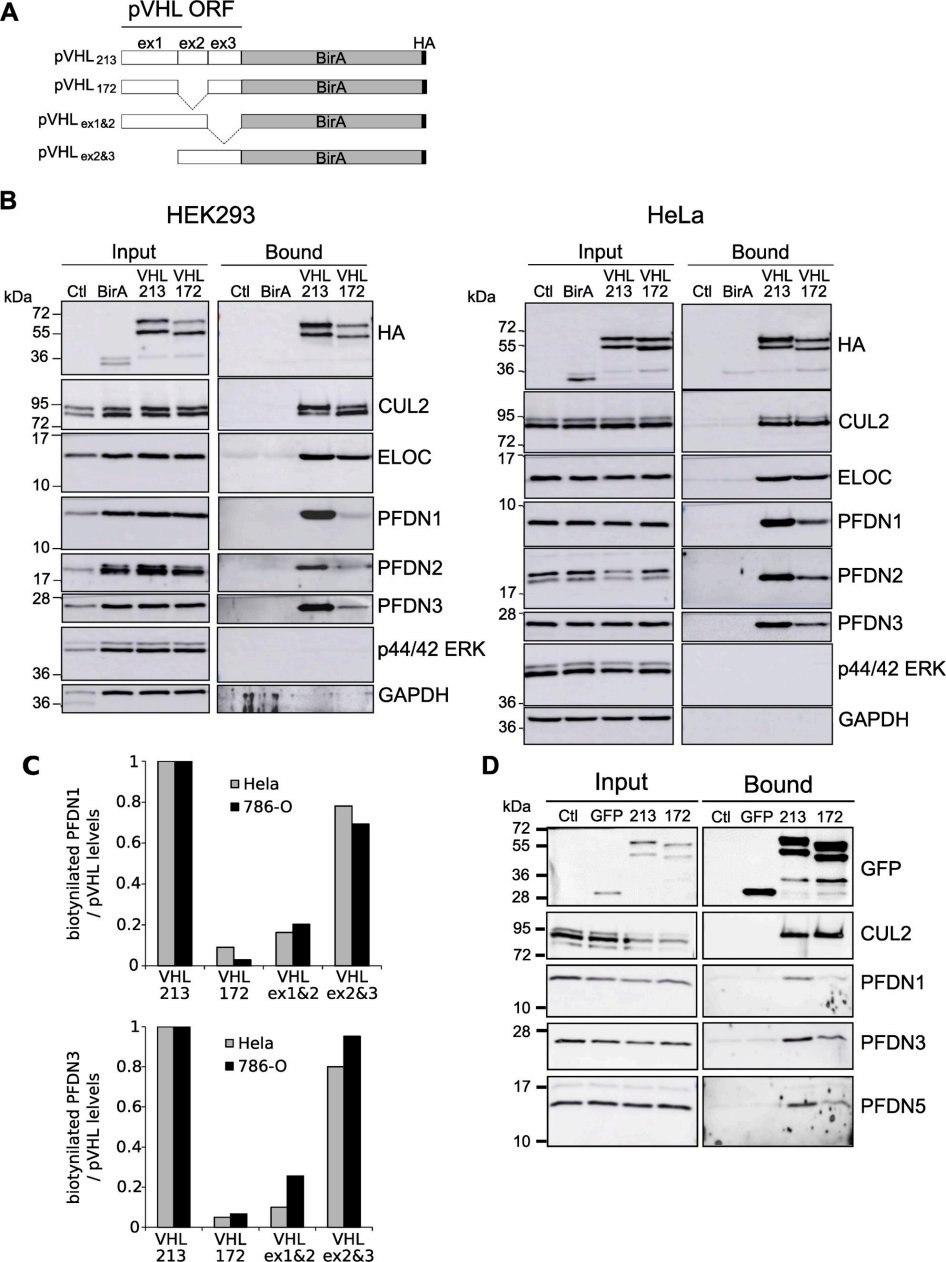
Prefoldin subunits PFDN1, PFDN2 and PFDN3 associate with pVHL in human cells. A) Scheme depicting the different BirA fusion proteins used in the BioID experiment (ex1, ex2 and ex3 refer to VHL exon1, exon2 and exon3-encoded polypeptides, respectively), B) Western blot analysis of PFDN1, PFDN2, PFDN3 and BirA fusion proteins in total protein extracts before (Input) and in fractions recovered after the Streptavidin affinity-chromatography column (Bound). Cullin 2 (CUL2) and Elongin C (ELOC) were used as positive controls whereas GAPDH and p44/42 ERK were used as negative controls. Ctl corresponds to untransfected control cells. Left: HEK293 cells and right HeLa cells. C) Quantification of the biotinylated prefoldin / pVHL expression levels to map the prefoldin binding site in pVHL ORF: truncated VHL213-BirA constructs expressing either all 3 exons (VHL_213_), exons 1+3 (VHL_172_), exons 1+2 (VHL_ex1&2_) and exons 2+3 (VHL_ex2&3_) were used in a BioID experiment in HeLa and 786-O cells. Histograms represent the mean relative levels of recovered PFDN1 (upper panel) and PFDN3 (lower panel) proteins. Full-length VHL213-BirA level was set as 1. D) HeLa cells expressing GFP, VHL213-GFP (213), VHL172-GFP (172) or control untransfected cells (Ctl) were lysed and immunoprecipitation was performed with GFP-trap. The whole cell lysates (Input) and immunoprecipitates (Bound) were analyzed by Western blot using anti-GFP, anti-CUL2, anti-PFDN1, anti-PFDN3 and anti-PFDN5 antibodies.

A BirA-based proximity biotinylation assay coupled to mass spectrometry (BioID) was used as a strategy to identify novel isoform-specific pVHL-interacting proteins. pVHL213-BirA and pVHL172-BirA fusion proteins were expressed as baits in HEK293 human cells (Fig 1A). Well characterized pVHL binding partners such as Cullin 2 and Elongin C were detected in the biotinylated pool of proteins co-eluted with both pVHL213 and pVHL172. Notably, the six PFDN subunits of the hexameric protein complex prefoldin were identified with pVHL213-BirA (Table 1). This result is consistent with the previously described interaction of pVHL with the PFDN3 subunit [29]. However, our data strongly suggests that the whole hexameric prefoldin complex may be critical for the regulation of pVHL.

**Table 1:** Identification of the whole prefoldin complex by mass spectrometry in streptavidin pulldowns in HEK293 cells expressing VHL213-BirA (see Materials and Methods for LFQ intensity calculation). LFQ intensities for prefoldin subunits are in light grey.

Gene name	Description	LFQ intensity BirA	LFQ intensity VHL-172-BirA	LFQ intensity VHL-213-BirA	LFQ intensity CTRL
CCT8	T-complex protein 1 subunit theta	2.35E+08	3.86E+10	7.42E+10	2.85E+07
CCT2	T-complex protein 1 subunit beta	5.32E+06	5.70E+09	1.91E+10	4.34E+06
CCT5	T-complex protein 1 subunit epsilon	2.17E+07	4.20E+09	1.61E+10	1.37E+07
CCT6A	T-complex protein 1 subunit zeta	2.50E+08	4.75E+09	1.83E+10	1.43E+08
TCP1	T-complex protein 1 subunit alpha	1.28E+09	5.98E+09	1.76E+10	1.16E+09
CCT3	T-complex protein 1 subunit gamma	9.10E+07	5.43E+09	1.69E+10	4.84E+07
CCT7	T-complex protein 1 subunit eta	6.69E+06	4.53E+09	1.53E+10	0.00E+00
CCT4	T-complex protein 1 subunit delta	7.29E+07	2.99E+09	1.11E+10	4.02E+07
CUL2	Cullin-2	0.00E+00	4.79E+08	5.54E+08	0.00E+00
**PFDN2**	Prefoldin subunit 2	0.00E+00	5.14E+07	8.73E+08	0.00E+00
**PFDN1**	Prefoldin subunit 1	0.00E+00	0.00E+00	9.67E+08	0.00E+00
**VBP1**	Prefoldin subunit 3	0.00E+00	0.00E+00	4.02E+08	0.00E+00
**PFDN6**	Prefoldin subunit 6	0.00E+00	0.00E+00	4.35E+08	0.00E+00
**PFDN4**	Prefoldin subunit 4	0.00E+00	0.00E+00	1.60E+08	0.00E+00
TCEB1 (EloC)	Transcription elongation factor B polypeptide 1 (Fragment)	0.00E+00	2.23E+08	2.85E+08	0.00E+00
**PFDN5**	Prefoldin subunit 5	0.00E+00	0.00E+00	1.51E+08	0.00E+00
RBX1	E3 ubiquitin-protein ligase RBX1	0.00E+00	0.00E+00	1.94E+07	0.00E+00

To validate whether this interaction is observed in different human cell lines, we monitored the presence of PFDN proteins in the biotinylated pool of pVHL-interacting proteins in three different cell lines HEK293, HeLa and 786-O after expression of pVHL213-BirA or pVHL172-BirA (Fig 1B and S1 Fig). As expected, Cullin 2 (CUL2) and EloC (ELOC) were immunodetected in the streptavidin-bound proteins in both HEK293 and HeLa cell extracts (Bound, Fig 1B and 1C) whereas negative controls (GAPDH, p44/42 ERK) were not. PFDN1, PFDN2 and PFDN3 proteins were immunodetected in the affinity-purification extracts obtained when pVHL213-BirA was expressed in both HEK293 and HeLa cells (Fig 1B). However, the signal corresponding to PFDN3 was lower in pVHL172-BirA streptavidin isolates for both cell lines. The signal corresponding to PFDN1 and PFDN2 proteins were even much lower in proteins found in pVHL172-BirA streptavidin extracts of HEK293 and HeLa cells compared to the pVHL213-BirA isoform (Bound, Fig 1B). The association of PFND1 and PFDN3 subunits with pVHL213-BirA was confirmed in streptavidin affinity-purification extracts obtained with 786-O cells but not in cells expressing pVHL172-BirA (S1 Fig). Consistently, PFDN1 and PFDN3 subunits were not recovered in the BioID screen with pVHL172-BirA fusion protein. PFDN2 was detected although with a ten times lower abundance than any PFDN subunit isolated with pVHL213-BirA (Table 1). As expected, PFDN subunits were not present in biotinylated pools of untransfected (Ctl) or BirA expressing controls (Fig 1B and S1 Fig). These observations implicates a robust and preferential attachment of the hexameric prefoldin complex to the pVHL213 isoform.

To confirm these results, we carried out a proximity-ligation-assay experiment. We used 786-O-pVHL213, a human renal 786-O cell line stably expressing Flag-HA-pVHL213 [4] and specific anti-HA, anti-PFDN1 and anti-PFDN3 antibodies to analyze a possible colocalization of Flag-HA-pVHL213 with PFDN1 and PFDN3. A representative experiment is shown on S2 Fig. In four independent experiments, the results showed a significant increase of the signal in 786-O-pVHL213 cells compared to the 786-O parental cell line for PFDN1 (11.0±4.8 dots/cell and 3.2±1.7 dots/cell, respectively; t-test: p value 0.04). A similar difference was observed in 786-O-pVHL213 cells compared to 786-O cells for PFDN3 (21.7±5.0 dots/cell and 7.5±5.6 dots/cell, respectively; t-test: p value 0.009), revealing a colocalization between pVHL213 and both PFDN1 and PFDN3.

The weaker interaction between prefoldin subunits and pVHL172 observed in HEK293 and HeLa cells or the absence of interaction in 786-O cells may suggest that part of the prefoldin binding site lies within the exon 2 encoded domain of pVHL213 that is missing in the pVHL172 protein isoform. Consistently, we used different truncated versions of pVHL213-BirA (i.e. pVHL213_ex1&2_-BirA and pVHL213_ex2&3_-BirA, Fig 1A) to monitor their association with the prefoldin complex and compared them to pVHL172 (i.e. exons 1&3). We observed that a prefoldin-pVHL interaction was likely established through the protein domain encoded by the junction between exon 2 and exon 3 but not through exon1-2-encoded one. (Fig 1C). Importantly, quantitative measurements of pVHL172 (exons 1&3) binding with PFDN1 and PFDN3 was four-five times lower than with the exons 2&3 containing version.

To confirm this result, we mutated the aa144-156 region of the pVHL ORF by replacing the hydrophobic stretch (GQPIFANITLPVY) by a more neutral stretch of amino acids (GQPSTSNSTSPVY, S3A Fig). The produced VHL213mut version was fused to BirA, expressed in HEK293 cells and used in a BioID experiment to measure its prefoldin binding capacity. As shown on S3B and S3C Fig, the VHL213mut-BirA fusion protein showed a strong reduction in its capacity to biotinylate PFDN1, PFDN3 and PFDN5 that was quite similar to what was observed with the exon-2 deleted VHL172 variant. Hence, these results demonstrate that the aa144-156 region, probably due to its hydrophobic nature, is critical for the prefoldin-pVHL213 interaction. Our results also show that alteration of the aa144-156 region of pVHL213 ORF reduced the CUL2 interaction which is essential for the E3 ligase VBC complex function.

To independently validate the specificity of the prefoldin-pVHL interaction, we immunoprecipitated GFP-tagged pVHL213 and pVHL172 expressed in HeLa cells. We analyzed the presence of prefoldin subunits in the immunoprecipitates by Western blot. As shown on Fig 1D, PFDN1, PFDN3, and PFDN5 were preferentially detected in the pVHL213 immunoprecipitates. Prefoldin subunits were absent in the GFP control. As a positive control, similar CUL2 levels were also clearly detected in both pVHL213 and pVHL172 immunoprecipitates as previously reported. This latter result confirms that at least three prefoldin subunits (PFDN1, PFDN3 and PFDN5) are physically associated to form a complex with the pVHL213 isoform suggesting the involvement of the whole cochaperone complex in regulating pVHL213 protein.

Eukaryotic prefoldin is a highly structurally conserved hetero-hexameric complex involved in protein folding. We showed in a previous paper that GFP-tagged pVHL213 fusion protein expressed in fission yeast formed small aggregates and large inclusions because pVHL213 is misfolded in the absence of its human cognate partners [26]. To support a role of prefoldin in regulating pVHL213 proteostasis through a physical interaction, we immunoprecipitated GFP-pVHL213 from whole cell lysates prepared from fission yeast cells in late exponential growth phase. We compared the protein-protein interactomes of GFP and GFP-pVHL213 by quantitative mass spectrometry (S4 Fig). All the proteins co-eluted with GFP or GFP-pVHL213 are listed in S1 Table. Analysis of the gene ontology annotations of the 436 proteins specifically immunoprecipitated with GFP-pVHL213 revealed an enrichment for protein complexes involved in protein folding like the TRiC/CCT chaperonin, known to interact with pVHL [32] (S4 Fig). Among them, as expected for misfolded intermediates or protein aggregates, the heat shock protein Hsp90/Swo1, the Hsp70/Ssa1 and Hsp40/Mas5 proteins were identified. Interestingly, three prefoldin subunits Pfd2, Pfd3 and Pfd5 were recovered only in the GFP-pVHL213 immunoprecipitate and not in the GFP control one. We thus decided to study the fate of the pVHL213 protein when expressed in the context of fission yeast prefoldin deletion mutants.

### Characterization of fission yeast prefoldin mutants

As we have identified all PFDN subunits as potential pVHL partners in human cells, we investigated the aggregation propensity of pVHL in the different fission yeast PFDN mutants. To date, only two prefoldin subunits have been identified as contributing to protein stability through studies of their deletion mutants in fission yeast. Pfd5 (SPBC215.02), also referred to as Bob1, controls sexual differentiation [25] and Pfd3 (SPAC3H8.07c), also referred to as Pac10, regulates pVHL stability [26]. The Open Reading Frames of *pfd1* (SPBC1D7.01), *pfd2* (SPAC227.10), *pfd4* (SPAC227.05) *and pfd6* (SPAC3A11.13) genes were identified in the fission yeast genome using the Pombase resource (https://www.pombase.org/) and we named the subunits Pfd1, Pfd2, Pfd4, and Pfd6 respectively, based on their sequence similarity with their human counterparts (S2 Table). In other species, prefoldin subunits have a high propensity to form coiled-coils structures. Each prefoldin subunit is formed by two alpha-helices present in the NH_2_ and COOH terminal parts separated by a β hairpin linker in the central region. As for prefoldin subunits found in other species, all six fission yeast Pfd ORFs shared this same structural organization. Each β hairpin linker consists of four or two short β strands for alpha and beta prefoldin subunits, respectively [19]. In accordance with this nomenclature, Pfd3 and Pfd5 encodes alpha-prefoldin subunits while Pfd1, Pfd2, Pfd4 and Pfd6 are beta-subunits (S5 Fig). The ORFs also showed substantial identities to other prefoldin subunits in other species (S2 Table).

To address the role of all prefoldin subunits in pVHL proteostasis in the *S*. *pombe* system, *pfd1*Δ to *pfd6*Δ deletion mutants were either obtained from other laboratories or prepared in our lab (*pfd1*Δ, see Materials and Methods).

Fission yeast *pfd1*Δ, *pfd2*Δ, *pfd4*Δand *pfd6*Δ mutants have never been characterized so far. Prefoldin regulates alpha-tubulin expression levels and consequently the microtubule (MT) network organization and function in other model organisms [16,22,23,31]. Consistently, Henkel *et al*. (2001) showed that fission yeast *pfd5*Δ cells were hypersensitive to the microtubule depolymerizing drug thiabendazole TBZ [25]. Thus, we postulated that other prefoldin mutants should exhibit MT-deficient phenotypes in fission yeast. Consistent with this hypothesis, all *pfd* mutants exhibited hypersensitivity to TBZ, especially alpha-subunit encoding mutants *pfd3*Δ and *pfd5*Δ, and cold sensitivity, a phenotype commonly observed for MT-deficient mutants [16,33] (S6A Fig). Other MT-deficient phenotypes have been also observed in all *pfd* mutants. First, in interphase, MT pushing forces are involved in nuclear centering [34]. Therefore, in MT organization deficient cells, interphase nuclei and nuclei undergoing segregation during anaphase were often asymmetric or off-centered (S6B Fig). Nuclear positions were observed in *pfd* mutant cells at 30°C but also at 20°C since this latter temperature may exacerbate MT-deficient phenotypes. Accordingly, nuclei were twice more frequently off-centered in all *pfd* mutants compared to wild-type cells and this phenotype was observed at both temperatures (S6C Fig). Secondly, due to spindle defects, mitotic segregating DNA was frequently stretched or unequally partitioned in such cells (archery bow and unequal segregation phenotypes, respectively, S6B Fig). In *pfd* mutants, we observed twice more frequently mitotic defects such as archery-bow like DNA structures and unequal chromosome partition in mitosis (S6D Fig). Third, using strains expressing GFP-tagged alpha-tubulin Atb2, we observed 3 to 6 MT bundles in interphase wild-type fission yeast cells. In contrast, *pfd1*Δ and *pfd3*Δ interphase cells showed reduced intensity of GFP-Atb2 labelled MT structures and a significant reduction in the number of MT bundles per cell (S6E and S6F Fig). Last, GFP-Atb2 expression level was monitored by Western blot. We consistently observed reduced expression levels in *pfd1*Δ and *pfd3*Δ strains (42% and 24% of wild-type levels, respectively; S6G Fig). We conclude that all prefoldin fission yeast mutants show altered MT phenotypes.

### An integrated cassette to homogeneously express pVHL in fission yeast

**Fig 2:**
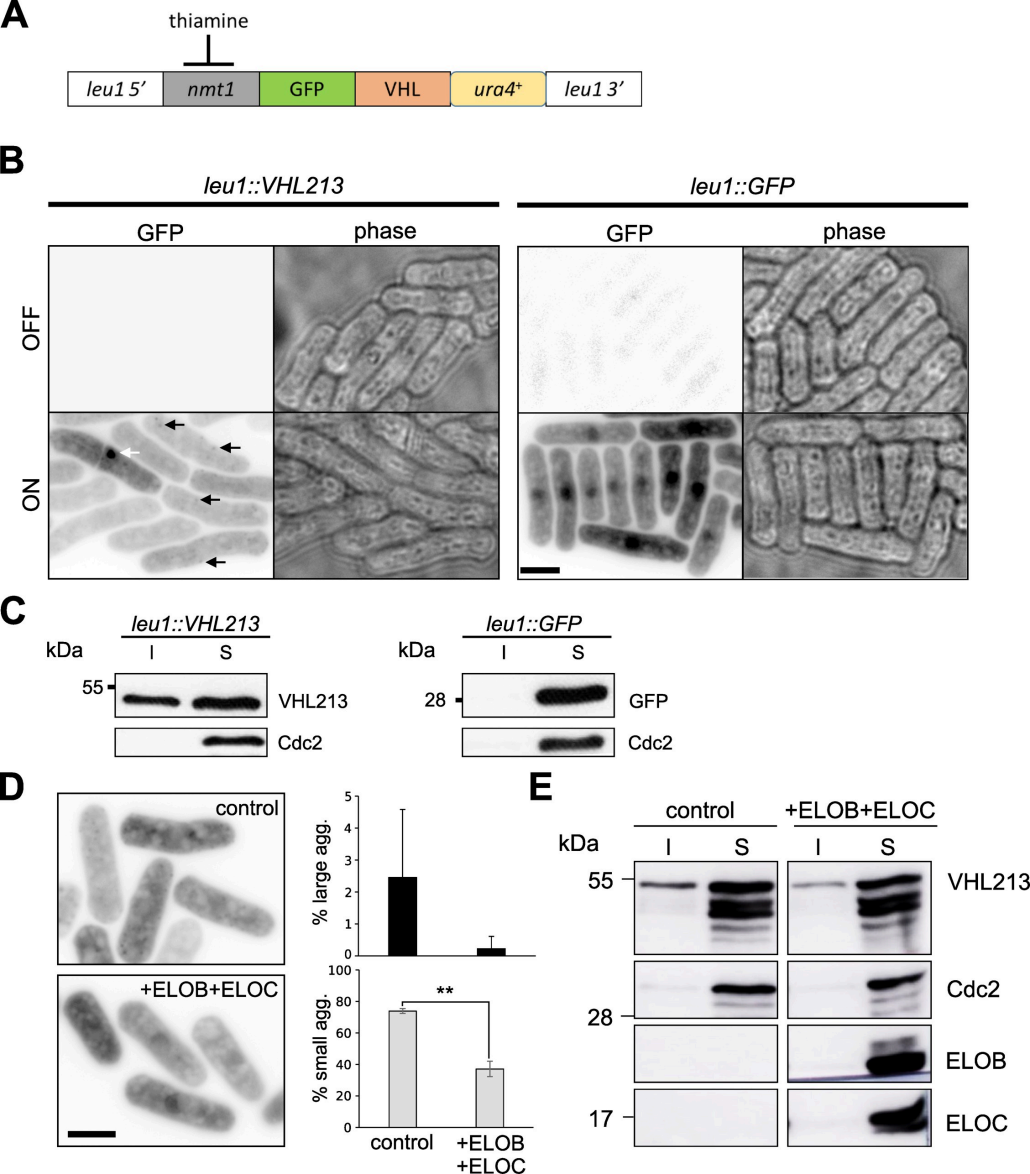
A conditional expression system to produce GFP-pVHL213 in fission yeast. A) Scheme of the integration cassette GFP-pVHL213 at the *leu1* locus, GFP-pVHL213 is under the thiamine-repressible promoter *nmt1*. B) Reversed fluorescent (left) or phase contrast (right) images of *leu1*::*VHL213* and *leu1*::*GFP* cells in the OFF (upper panels) or ON (lower panels) conditions. White and black arrows depict large and small aggregates, respectively. C) Western blot analysis of GFP-pVHL213 or GFP expression (ON condition) in *leu1*::*VHL213* and *leu1*::*GFP* cells, respectively: Soluble (S) and Insoluble (I) fractions are shown. Cdc2 was used as a loading control. D) Aggregation of GFP-pVHL213 was compared between *leu1*::*VHL213* cells transformed with empty control vectors (v1/v2) or v1-ElonginB and v2-Elongin C (+ELOB+ELOC) expressing plasmids. Left: reversed fluorescent images and right: histograms representing the percentage of large (upper panel) and small (lower panel) aggregate-containing cells (mean±s.d. from three independent experiments; **, p<0.01, Student t test). E) Western blot analysis of GFP-pVHL213 (VHL213), Elongin B (ELOB), Elongin C (ELOC) in cells: Soluble (S) and Insoluble (I) fractions are shown. Cdc2 was used as a loading control. Bars: 5 μm.

To produce a homogeneous expression level of pVHL in all cells, we integrated a GFP-tagged VHL ORF under the control of the strong *nmt1* thiamine-repressible promoter at the *leu1* locus. Hence, we produced a *leu1*::*VHL213* strain and a *leu1*::*GFP* strain as a control (Fig 2A). After overnight overexpression, homogeneous aggregation patterns of small highly dynamic aggregates were observed in the yeast *leu1*::*VHL213*. Thus, misfolded pVHL213 expressed in yeast from a single locus was incorporated into small aggregates (approximately 30 per cell, black arrows, Fig 2B). We rarely observed large aggregates in the *leu1*::*VHL213* cells (<0.5%, white arrow, Fig 2B). The aggregated fraction of pVHL213 protein was detected in the insoluble fraction by Western blot analysis (I, Fig 2C). In contrast, GFP did not form any aggregate and remained in a diffuse soluble form (Fig 2B and 2C).

It has been proposed that pVHL is only properly folded after being bound to its cognate partners Elongin B and Elongin C [35]. To support the idea that aggregation of pVHL in yeast was due to misfolding, we hypothesized that co-expression of human Elongin B and Elongin C would suppress pVHL aggregation. Indeed, co-expression of human EloB/EloC binding partners efficiently restored pVHL solubility as the proportion of cells containing pVHL aggregates diminished from 80 to 40% and, concomitantly, the insoluble protein fraction decreased (Fig 2D and 2E).

**Fig 3:**
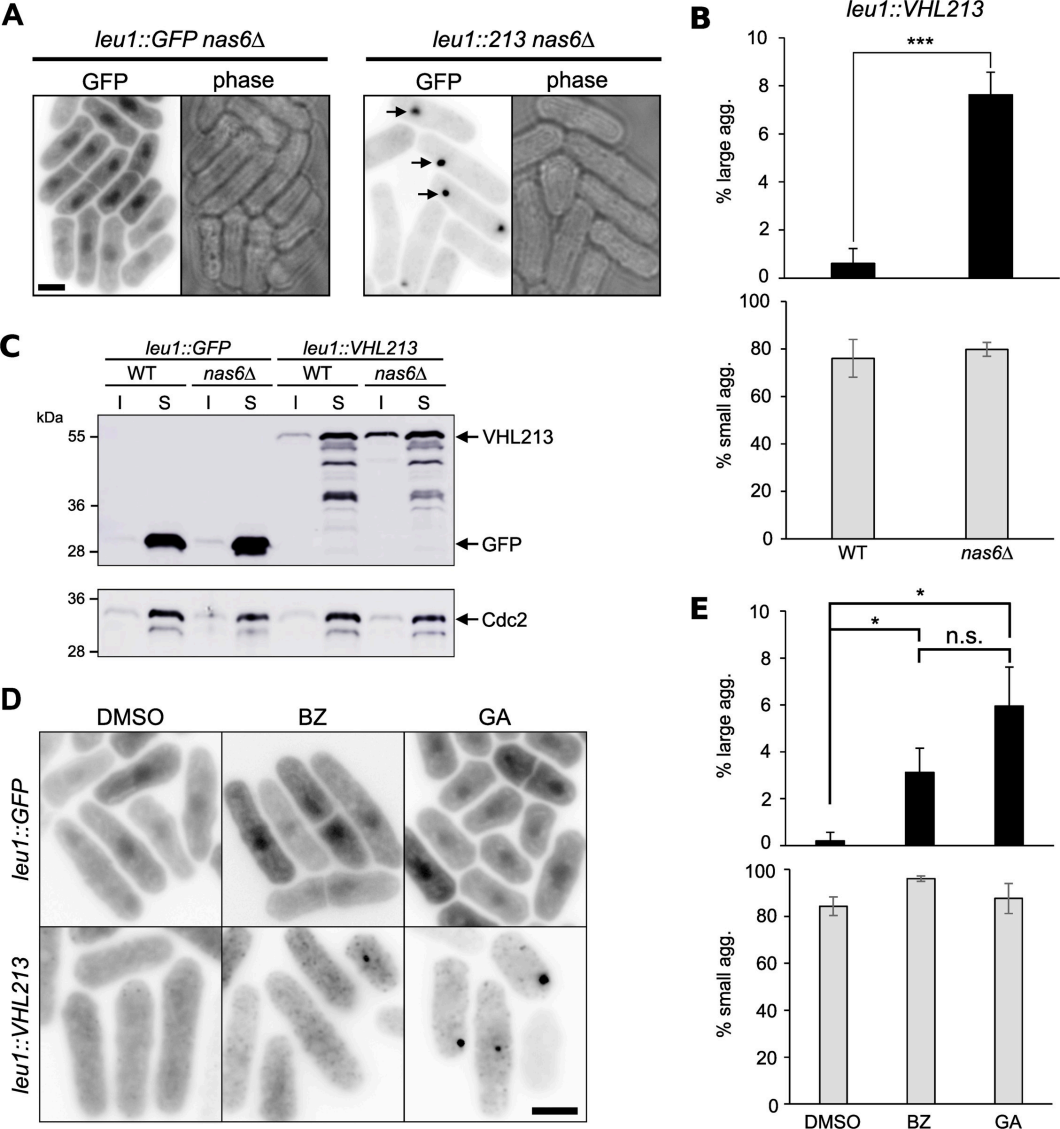
Inhibition of the proteasome or of the Hsp90 chaperone stimulates GFP-pVHL213 aggregation. A) Reversed fluorescent (left) or phase contrast (right) images of *leu1*::*GFP nas6*Δ and *leu1*::*VHL213 nas6*Δ cells. Arrows depict large GFP-pVHL213 aggregates. B) Percentage of large (upper panel) and small (lower panel) aggregate-containing cells in *leu1*::*VHL213* (WT) and *leu1*::*VHL213 nas6*Δ (*nas6*Δ) cells (mean±s.d. from three independent experiments; ***, p<0.001, Student t test). C) Western blot analysis of GFP or GFP-pVHL213 expression in *leu1*::*GFP* (WT and *nas6*Δ) and *leu1*::*VHL213* (WT and *nas6*Δ) backgrounds: Soluble (S) and Insoluble (I) fractions are shown. Cdc2 was used as a loading control. D) Reversed fluorescent images of *leu1*::*GFP* and *leu1*::*VHL213* cells cultured in the presence of DMSO (vehicle), Bortezomib (BZ) or Geldanamycin (GA). E) Percentage of large (upper panel) and small (lower panel) aggregate-containing cells in *leu1*::*VHL213* cells treated with DMSO, BZ or GA (mean±s.d. from three independent experiments; n.s., not significant; *, p<0.5, Student t test). Bars: 5 μm.

We reasoned that aggregates of misfolded pVHL proteins could be degraded by the ubiquitin-proteasome system. To validate this hypothesis, we out-crossed the strains expressing either GFP or GFP-pVHL213 to the proteasome-deficient mutant *nas6*Δ. We observed a higher aggregation pattern for *leu1*::*VHL213* in this mutant background (87.4±3.7% of aggregate containing cells), especially 7.6±0.9% of cells showing large aggregates indicated by arrows in Fig 3A, compared to 76.6±8.5% aggregate containing cells in wild-type cells, including 0.6±0.6% of cells with large aggregates (Fig 3B). We also observed an increase of pVHL213 levels in the insoluble fraction of *leu1*::*VHL213 nas6*Δ cell extracts (Fig 3C). In this genetic background, GFP remained soluble as in wild-type cells. To confirm our hypothesis of proteasome-mediated degradation of pVHL213 aggregates, we treated *leu1*::*VHL213* cells with the proteasome inhibitor Bortezomib (BZ, Fig 3D). We consistently observed an increased abundance of small intense aggregates as well as large aggregates in BZ-treated cells (3.7% for *leu1*::*VHL213*, Fig 3E).

In budding yeast, it has been shown that aggregation of misfolded proteins was increased when the Hsp90 chaperone function is inhibited [36]. Furthermore, Hsp90 has been shown to be directly involved in pVHL degradation in the budding yeast [37]. A further confirmation of pVHL213 propensity to aggregate was obtained by treating cells with the Hsp90 chaperone inhibitor Geldanamycin (GA). We observed aggregation stimulation in GA-treated *leu1*::*VHL213* but not in *leu1*::*GFP* cells (Fig 3D and 3E). With thiamine-controlled promoter and BZ or GA addition in the culture medium, we therefore set up a conditional pVHL aggregation system for pVHL213 that could be further used to investigate prefoldins’ effects on the E3 ligase folding.

### The prefoldin complex protects pVHL213 from aggregation

**Fig 4:**
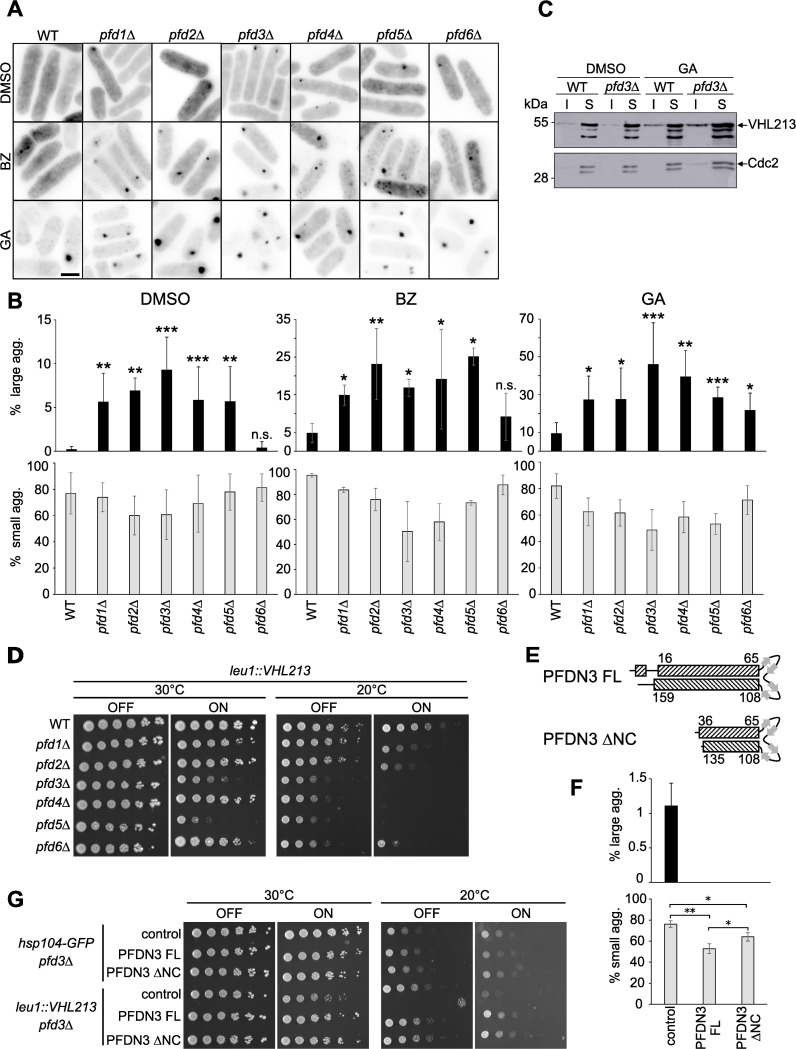
Aggregation and toxicity of pVHL213 are increased in prefoldin mutants. Cells of *leu1*::*VHL213* (WT) and *leu1*::*VHL213* prefoldin mutant as indicated were expressing GFP-pVHL213. A) Reversed fluorescent images of the different strains expressing GFP-pVHL213 treated with DMSO, BZ or GA. Bar: 5 μm. B) Histograms representing the percentage of large (upper panels) and small (lower panels) aggregate-containing cells in DMSO, BZ and GA treated cells (mean±s.d. from at least three independent experiments, *, p<0.05; **, p<0.01; ***, p-value<0.001; WT *vs pfd* mutant for each condition, Mann-Whitney test), C) Western blot analysis of GFP-pVHL213 expression in WT and prefoldin *leu1*::*VHL213 pfd3*Δ mutant in the presence of GA or DMSO as a control: Soluble (S) and Insoluble (I) fractions are shown. Cdc2 was used as a loading control. D) Serial dilutions (1:5) of the indicated strains were spotted on EMM plates in the OFF or ON conditions and incubated for 5 days at 30°C and 7 days at 20°C as indicated. E) Scheme representing the secondary structures of full-length (FL) or mutated (ΔNC) human PFDN3 protein: α-helices and β-strands are represented by dashed boxes and grey arrows, respectively. Numbers refer to the amino acids in the full-length PFDN3 protein. F) Histograms representing the percentage of large (upper panel) and small (lower panel) aggregate-containing cells in *leu1*::*VHL213 pfd3*Δ cells after expression of the full-length (FL) or mutated (ΔNC) human PFDN3 protein or in control cells transformed with empty vector (mean±s.d. from three independent experiments), G) Serial dilutions (1:5) of the indicated strains were spotted on EMM plates in the OFF or ON conditions and incubated for 5 days at 20°C and 30°C as indicated. *Hsp104-GFP pfd3*Δ transformants were used as a wild-type control.

To address the role of prefoldin interaction with pVHL, we analyzed pVHL aggregation phenotype in all six prefoldin deletion mutants. We microscopically observed that all *leu1*::*VHL213 pfd* mutant strains showed a significantly higher proportion of large aggregate-containing cells (up to 10.3% in *pfd3*Δ mutant) compared to 0.1% in wild-type background (Fig 4A and 4B). Therefore, the absence of any single prefoldin subunit stimulated pVHL aggregation. This suggests that the whole prefoldin complex is required to maintain a pool of soluble pVHL. This phenotype was strikingly exacerbated when cells were treated with GA or BZ to stimulate aggregation. In the presence of BZ, the percentage of cells containing large aggregates strikingly increased from 7% (*pfd6*Δ) up to 37% in *pfd2*Δ mutant compared to 4% in wild-type cells. A similar aggregation stimulation was observed in GA-treated cells showing 27% to 61% of large aggregate containing cells in *pfd* mutants, contrasting with the 6.9% seen in wild-type cells (Fig 4A and 4B).

The expression levels of GFP-pVHL213 were similar in WT and *pfd3*Δ cells (Fig 4C). However, it was difficult to observe a significant increase of pVHL immunoblot signal in the insoluble fraction of protein extracts prepared from *pfd3*Δ cells (DMSO, Fig 4C) even though the proportion of aggregate containing cells was higher (Fig 4B). When cells were treated with GA, a rather modest increase in the insoluble fraction of pVHL213 could be observed for the *pfd3*Δ mutant (GA, Fig 4C) compared to the increase of aggregate containing cells. (Fig 4B).

Since we observed that fission yeast *pfd* mutant cells were cold sensitive (S6 Fig), we assayed wild type and *pfd* mutant growth on solid medium while cells were overexpressing pVHL213 at both 30°C and 20°C. Interestingly, overexpression of GFP-VHL decreased growth in *pfd* mutants but not in WT cells. This phenotype was exacerbated at 20°C (Fig 4D), even in the OFF condition for some mutants when the expression levels of pVHL213 is repressed. Thus, overexpression of pVHL213 is toxic in *pfd* mutant backgrounds whereas it is not in wild type cells.

A possible explanation for increased pVHL213 aggregation would be that *pfd* mutants were subjected to a general proteotoxic stress. To address this question, we first quantified the Hsp70 steady state levels in prefoldin mutants at 30°C (S7A Fig). No significant difference was observed between any of the prefoldin mutants and the wild type strain. After a 2-hour heat shock at 37°C (S7B Fig), no significant difference in the increase in Hsp70 amounts (Hsp70 at 37°C/Hsp70 at 30°C) was measured (1.83, 1.73 and 1.62 for WT, *pfd1*Δ and *pfd3*Δ strains, respectively; mean of three independent experiments; Student t tests). Altogether, these results suggest that *pfd* mutants exhibit a wild type response to heat regarding Hsp70 protein levels. Second, we studied the growth phenotypes of prefoldin mutants after exposure to a 50°C heat shock for 15 minutes (S7C Fig). There was no growth difference between wild type, *pfd* mutant strains and the thermotolerance-deficient *hsp104*Δ strain [38]. In contrast, the *mas5*Δ strain which exhibits a high Hsf1 constitutive activity was able to partially grow [39]. When cells were incubated for 45 minutes at 37°C before being exposed to 50°C, wild type as well as *pfd* mutant strains could recover growth but not the thermotolerance deficient *hsp104*Δ strain as previously reported [38]. This result suggests that *pfd* mutants show a similar Hsf1-dependent response to heat shock as wild type cells. Third, we examined the presence of Hsp104-GFP dots that are markers of damaged or misfolded protein aggregates (S7D Fig). We could not observe a significant difference of Hsp104-GFP aggregation between wild-type and *pfd3*Δ cells at 30°C and even at 20°C (S7D Fig). We concluded that prefoldin mutants are unlikely subjected to a permanent proteotoxic stress but rather specifically promote the aggregation of pVHL213. Consistently, we monitored the growth phenotypes of prefoldin mutants on plates containing the proline analog azetidin-2-carboxylic acid (AZC) which promotes general protein misfolding (S7E Fig). There was no growth difference between wild type and *pfd* mutant strains compared to the AZC-sensitive *ppr1*Δ strain [40]. This suggests that *pfd* mutants are not sensitive to misfolding of nascent proteins caused by the AZC compound [41]. Furthermore, we studied the levels of total ubiquitinated proteins by Western blot after GST pulldown on GST-TUBE-loaded glutathione-agarose beads (see Materials and Methods, S7F Fig). No significant difference was observed between *pfd1*Δ or *pfd3*Δ mutants and the wild type strain, suggesting a similar content of ubiquitinated proteins in wild type and *pfd* mutant strains. Finally, we studied the behavior of the GFP protein in a *leu1*::*GFP pfd2*Δ strain (S7G Fig). In the absence or in the presence of BZ, no GFP aggregate was detected. Thus, the *pfd2*Δ mutation did not trigger aggregation of a soluble protein. More specifically regarding pVHL, we examined the aggregation pattern of the *leu1*::*VHL172 pfd2*Δ strain. In this strain, we expressed the exon2-deleted VHL isoform that exhibits a low propensity to aggregation due to the lack of an aggregation-prone region [26]. In wild-type or prefoldin mutant backgrounds, pVHL172 expressing cells never exhibited aggregates. However, in the presence of BZ, a similar pattern of pVHL172 aggregation was observed in both wild-type and prefoldin mutant strains. In support of this, prefoldin subunits were poorly recovered in the BioID screen with VHL172-BirA, which also lacks the exon2-exon3 junction that binds the prefoldin complex (Table 1, Fig 1).

Another possible explanation for increased pVHL213 aggregation would be an indirect effect of a *pfd* mutation-dependent MT network deficiency. To address this issue, we tested two different TBZ concentrations in the culture medium. As previously reported [42], both concentrations promoted a dose-dependent alteration of the MT network in fission yeast GFP-Atb2 expressing cells (S8A Fig). When *leu1*::*VHL213* cells were incubated with these TBZ concentrations, no significant effect on the pVHL213 aggregation pattern was observed (S8B and S8C Fig). To confirm this result, we also monitored pVHL213 aggregation in the kinesin microtubule motor-encoding gene deletion mutant *tea2*Δ which has been reported to exhibit a highly penetrant abnormal MT cytoskeleton phenotype [43]. In *leu1*::*VHL213 tea2*Δ cells, no significant alteration of aggregation pattern of pVHL213 was observed compared to control cells (S8B and S8C Fig).

To test whether the prefoldin-dependent pVHL213 aggregation suppression is conserved, we conditionally overexpressed the full-length human PFDN3 protein (Fig 4E) in a *pfd3*Δ background together with pVHL213 expression. Consecutively, human PFDN3 expression suppressed aggregation and reduced growth as well (Fig 4F and 4G). Thus, the role of PFDN3 in preventing pVHL213 aggregation is conserved by its human counterpart.

To support the idea that the chaperone function of the prefoldin complex is involved in pVHL213 aggregation and toxicity in yeast, we expressed PFDN3 ΔNC, a truncated version of human PFDN3, which alters the prefoldin chaperone function. Based on the work of Simons *et al*. (2004), we removed NH_2_-terminal and COOH-terminal extremities of PFDN3 (20 and 24 amino acids of the NH_2_ and COOH terminal alpha-helices, respectively), the tips of the tentacles that are involved in substrate binding [19,44]. The ΔNC construct maintained the presence of the central β hairpin making coiled-coil formation of truncated PFDN3 still possible. The PFDN3 ΔNC version was unable to suppress pVHL213 aggregation as efficiently as the full-length protein (lower panel, Fig 4F). This result suggests that the chaperone function of PFDN3 is critical to prevent pVHL213 aggregation. However, PFDN3 ΔNC was still retaining partial function when compared to the empty vector control (upper and lower panels, Fig 4F). Consistent with this, expression of the PFDN3 ΔNC mutant was still able to rescue the growth defect of *pfd3*Δ cells overexpressing VHL213 (ON, Fig 4G).

### Role of the prefoldin complex in pVHL stability in human cells

**Fig 5:**
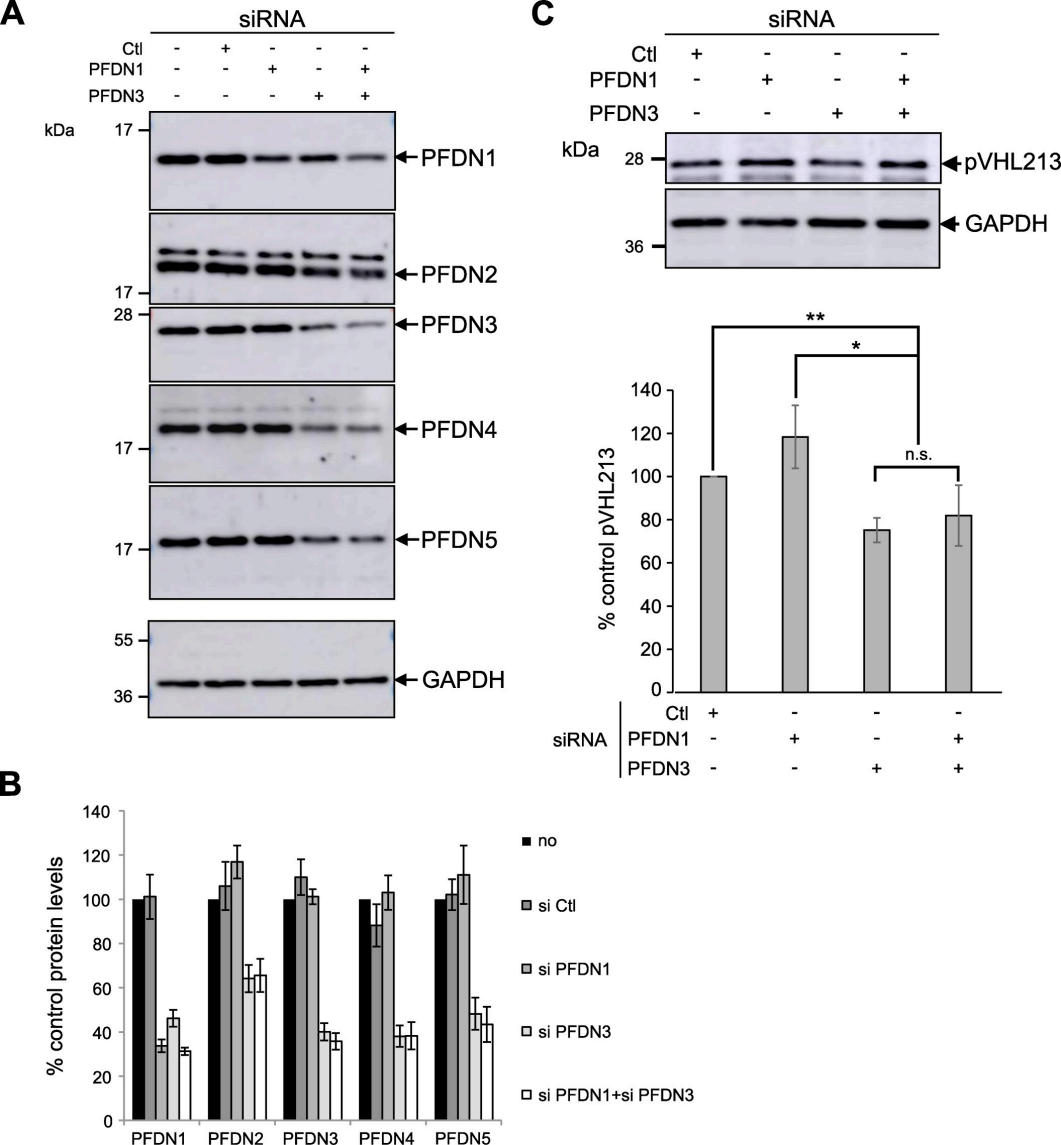
Downregulation of the prefoldin complex affects pVHL213 stability in human cells. A) Western blot analysis of PFDN1, PFDN2, PFDN3, PFDN4 and PFDN5 expression levels after siRNA targeting PFDN1, PFDN3 or PFDN1+PFDN3 in HEK293 cells. GAPDH was used as a loading control. B) Histogram representing the amount of PFDN subunits levels in siRNA experiments (mean±s.d. from at least three independent experiments). “Si Ctl” represents cells transfected with mock siRNA. C) Up: Western blot analysis of pVHL213 expression after siRNA of PFDN1, PFDN3 or PFDN1+PFDN3 in HEK293 cells. Bottom: Histogram representing the pVHL213 levels in PFDN siRNA experiments (mean±s.e.m. from at least three independent experiments). *, p-value<0.05; **, p-value<0.01. Mann-Whitney test.

Since prefoldin is conserved in human cells (S2 Table), we investigated a potential role of prefoldin in pVHL stability by siRNA-mediated prefoldin subunit down-regulation. Selected siRNA targeting PFDN1 and PFDN3 were transfected individually or together (Si PFDN1 +Si PFDN3) in HeLa cells. PFDN3 silencing revealed that other PFDN subunits were also down-regulated (PFDN1, PFDN2, PFDN4 and PFDN5; Fig 5A and 5B). This down-regulation can be attributed to a protective effect of prefoldin complex formation against proteasome-mediated degradation of individual subunits, as previously proposed [45,46]. However, the PFDN1 siRNA efficiently reduced PFDN1 protein levels but did not affect the level of other PFDN subunits. Thus, in contrast with previous reports, PFDN1 siRNA did not contribute to a protective effect on the prefoldin complex in our experiments. Furthermore, the combination of PFDN1+PFDN3 siRNA did not exacerbate the global prefoldin down-regulation, suggesting that the protective effect against proteasomal degradation was solely due to the presence of PFDN3.

We assessed whether siRNA-mediated prefoldin down regulation altered the tubulin network. Using immunofluorescence experiments, we consistently and reproducibly detected aberrant tubulin network organization in PFDN1, PFDN3 and PFDN5 siRNA treated cells (S9 Fig). In control cells (untreated or control siRNA treated cells), the microtubules form a network of dense fibrils extending towards the plasma membrane. In contrast, in PFDN siRNA treated cells, the microtubule network was frequently concentrated either around the nucleus or at the plasma membrane and showed weak fibrillar structures in the cytoplasm. In addition, a two fold increase in late cytokinesis figures was observed in PFDN1 and PFDN3 siRNA treated cells. The MT-deficient phenotypes of PFDN siRNA were rather diverse for individual cells. But, altogether, these observations suggest that any PFDN down-regulation that we tested promoted an unambiguous MT network alteration, correlated with a reduced expression of the targeted PFDN analyzed by Western blot.

When we performed PFDN3 siRNA in HeLa cells, we observed a modest but significant 20–25% decrease of the pVHL213 steady-state levels (Fig 5C). This effect was not detected in PFDN1 siRNA samples and the combination of PFDN1+PFDN3 siRNA did not further decrease pVHL213 protein levels (Fig 5C). Thus, the prefoldin complex is critical to maintain pVHL213 stability but this effect likely required a full knock-down of the complex.

In addition, we studied the pVHL172 levels in siRNA experiments. We observed that neither the PFDN1 nor the PFDN3 knock-down significantly altered pVHL172 levels. However, the double siRNA (PFDN1+PFDN3) promoted a noteworthy decrease (S10 Fig). Since the PFDN1+PFDN3 double knock-down did not exacerbate the whole prefoldin complex down-regulation compared to PFDN3 single knock-down (Fig 5A), the observed effect on pVHL172 levels remains to be explored.

As PFDN silencing might promote an indirect transcriptional down-regulation of the *VHL* gene, the effect of the silencing of PFDN1, PFDN3 or PFDN1+PFDN3 on *VHL* expression in HeLa cells was assessed by RT-qPCR analysis. The experiment showed that none of PFDN siRNA conditions reduced the expression of either the full length (VHL213) or the exon2-deleted (VHL172) *VHL* variants whereas, as expected, PFDN1 and PFDN3 transcripts were greatly reduced 48 hours after PFDN1 and PFDN3 siRNA transfection, respectively (S11 Fig).

Finally we could not rule out that prefoldin subunits might be substrates of the pVHL213-containing VBC E3 ligase complex. This may impact the expression levels of PFDN subunits depending on the pVHL status in human cell lines. To address this issue, we monitored PFDN1 and PFDN3 expression levels in 786-O cell lines that showed different pVHL status (the parental 786-O VHL-/-, the 786-O-pVHL213 and 786-O-pVHL172 cell lines stably expressing pVHL213 or pVHL172, respectively) [4]. We observed no difference in PFDN1 and PFDN3 expression levels in pVHL213- expressing cells whereas expression levels of the known HIF2α VBC E3 ligase target was greatly diminished (S12A and S12B Fig). We observed a modest decrease of PFDN3 only in the 786-O-pVHL172 cells. However the E3 ligase activity of pVHL172 has not been demonstrated so far. Thus, this effect on PFDN3 levels remains to be elucidated. We confirmed these results by assaying PFDN1 and PFDN3 expression levels after transient transfection of VHL-expressing plasmids into HEK293 cells. Overexpression of pVHL213 may promote a VBC-dependent degradation of PFDN1 and/or PFDN3 if these prefoldin subunits are targeted by the E3 ligase complex. As shown in S12C Fig, the expression levels of PFDN1 and PFDN3 were not altered in cells expressing either wild type VHL213 (VHL213 wt), a mutated VHL213 deficient for prefoldin binding (S3 Fig, VHL213 mut) or VHL172. Thus, overexpression of pVHL213 did not trigger PFDN1 and PFDN3 degradation. Alternatively, we did neither observe that VHL213 expression increased PFDN1 and PFDN3 expression levels, ruling out a possible stabilizing effect of VHL213 binding on prefoldin subunits.

To further confirm these results, we examined a possible pVHL213-dependent ubiquitination of prefoldin subunits. To address this issue, we used the parental 786-O cell line (VHL-/-) as a control and stably transfected 786-O cells expressing either pVHL213 or pVHL172. We pulled-down total ubiquitin proteins using the GST-TUBE approach (S13A Fig). We verified that the HIF2α and the p21^CIP1^ proteins, known to be ubiquitinated, were recovered in the GST-TUBE fractions as multiple bands (S13B and S13G Fig). The HIF2α transcription factor is targeted in part by pVHL213 dependent VBC E3 ligase complex and consistently less HIF2α was recovered in 786-O-pVHL213 cells (S13B Fig). However, only very faint and single PFDN1 or PFDN3 bands (migrating as non ubiquitinated forms) could be detected in the GST-TUBE pull-down fractions, in pVHL expressing as well as in parental 786-O cells (S13E and S13F Fig, respectively). These results likely rule out the hypothesis of a pVHL-dependent PFDN ubiquitination.

### Analysis of the expression levels of PFDN3 in clinically relevant samples

**Fig 6:**
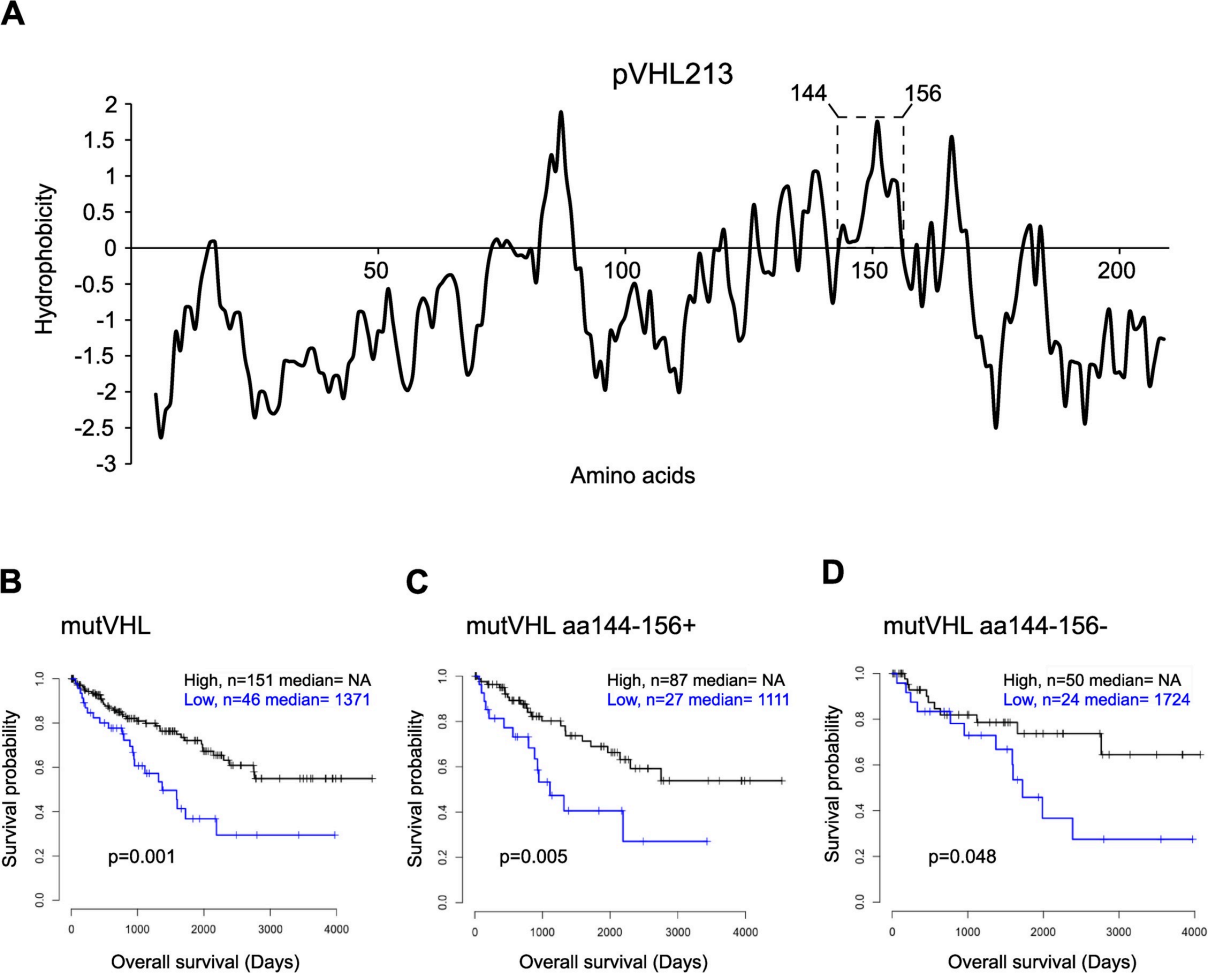
Expression levels of PFDN3 correlates with survival of ccRCC patients with mutated VHL. A) Kyte-Doolittle hydrophobicity plot for the whole pVHL213 protein, dashed inset shows the aa144-156 hydrophobic peak, B-D) Analyses by Kaplan-Meier curves to report overall survival of patients with High (grey line) or Low (blue line) PFDN3 expression levels extracted from the TCGA database: B) patients with mutated VHL (mutVHL, n = 197), C) mutVHL patients with an intact aa144-156 region (mutVHL aa144-156+, n = 114), D) mutVHL patients without aa144-156 region (mutVHL aa144-156-, n = 74). P values are indicated below the curves.

Our results in Fig 1 suggest that the prefoldin complex may preferentially bind to pVHL region encoded by the junction between exon 2 and exon3. In this region, the aa144-156 showed a prominent hydrophobic peak (S3A Fig and inset in Fig 6A) that might be recognized by the prefoldin complex and thus protected it from surface exposure. Indeed, in S3 Fig, we demonstrated that aa144-156 are critical for prefoldin-pVHL213 binding.

In Fig 5A, we showed that lowering PFDN3 expression levels had an impact on the amount of the whole prefoldin complex. It suggests that low PFDN3 levels correlate to a significant loss of the whole functional prefoldin complex in cells. To investigate the relationship between PFDN3 expression and disease progression, the Kaplan-Meier method was used to evaluate the patients’ overall survival relative to PFDN3 expression in ccRCC tumors. In TCGA database (https://www.cancer.gov/about-nci/organization/ccg/research/structural-genomics/tcga), we selected patients harboring either wild-type (n = 223) or mutated (n = 197) VHL. For wild-type VHL patients, no significant difference in overall survival was observed regarding low (n = 48) versus high (n = 175) levels of PFDN3 expression (p = 0.26). Thus, we considered the hypothesis that prefoldin expression levels might be critical mostly for mutated VHL as some of these VHL variants are likely unstable. Therefore, we focused on n = 197 ccRCC patients with mutated VHL (mutVHL) and found that the 46 patients with the lowest expression levels of PFDN3 had the worst survival (p = 0.001, Fig 6B). Next, we considered patients for which the putative prefoldin binding site (aa144-156 region) was unaffected (mutVHL aa144-156+, n = 114) and those with mutant VHL which had lost this region due to premature upstream STOP codon, frameshift or splicing mutations (mutVHL aa144-156-, n = 74). In this context, we found that the expression levels of PFDN3 was still relevant for patients’ survival harboring the aa144-156 region (mutVHL aa144-156+). For these patients, those with the lowest PFDN3 expression levels (n = 27) had the worst survival (p = 0.005, Fig 6C). In contrast, for mutVHL aa144-156- patients, the low (n = 24) versus high (n = 50) PFDN3 expression levels did not show a significant difference regarding patients’ survival (p = 0.048, Fig 6D).

Mutant pVHL that could no longer bind to the prefoldin complex are expected to be misfolded because they could not be loaded by the prefoldin complex onto TRiC/CCT chaperone complex. Aggregation and subsequent degradation of proteins usually occurs because hydrophobic stretches of amino acids are exposed during protein synthesis. It is generally accepted that the main role of chaperones is to prevent aggregation by binding to hydrophobic amino acid stretches thereby inhibiting their surface exposure. These results suggest that low PFDN3 expression in ccRCC has a strong negative prognostic impact in patients harboring VHL mutation with intact aa144-156 region.

## Discussion

In this study, we showed that the hexameric prefoldin complex is essential to the stabilization of pVHL. Based on the essential role of the prefoldin complex in the delivery of actin and tubulin to the chaperonin TRiC/CCT complex [19–21] we believe that we can extend this molecular function to the stabilization of VHL through a similar mechanism. This interaction-mediated substrate stabilization is likely critical to facilitate VHL folding in various VHL-related diseases [37,47]. We mapped this protein-protein interaction interface at the exon2-exon3 junction of pVHL where several frequent pathologic mutations have been documented [48,49]. The prefoldin-assisted protein folding process might even be more influential in high grade tumors, when protein quality control is known to be less efficient. In agreement with a role of prefoldin for loading pVHL onto TRiC/CCT, all eight subunits of the TRiC/CCT chaperonin complex have been recovered in both the human BioID screen and in the fission yeast immunoprecipitates.

Although new therapies have been successfully proposed in recent years (anti-angiogenic or immunomodulatory compounds), resistance mechanisms develop with disease progression in many cases of ccRCC. Thus, current therapies for VHL-related diseases remain ineffective in the long term, calling for new therapeutic avenues. Among the high number of reported VHL mutations, many are uncharacterized to date, preventing these new therapies from being developed. Many mutations in VHL Open Reading Frame may alter the precise folding steps leading to a fully functional native protein. Therefore, the understanding of pVHL folding steps is crucial. Previous studies already highlighted the importance of a chaperone complex, TRiC/CCT, which transfers folded pVHL to the E3 ligase complex VBC [32,37,47,50]. Unbound pVHL has been shown to be degraded by the proteasome thus limiting the cellular content of functional pVHL [35]. Analysis of PFDN3 expression profile in ccRCC patients, limited to missense VHL mutants, showed that a low expression level correlates with poor survival (Fig 6). We propose that low levels of PFDN3, which promote destabilization of the whole prefoldin complex (Fig 5A), could reduce the cellular pool of functional pVHL by limiting its folding efficiency and thereby exacerbating the *VHL* mutation phenotype. Consistently, Kim *et al*. reported that PFDN3 overexpression and shRNA knockdown increased or diminished pVHL expression levels, respectively. They showed an impact on metastasis in a mouse model after injection of melanoma cells *in vivo*. However, these authors proposed the existence of a TRiC/CCT-independent function of PFDN3 [30]. Albeit this function is not mutually exclusive with a prefoldin complex dependent one, it suggests that PFDN3 may act on pVHL stability through different mechanisms.

Here we have characterized the interaction between the pVHL protein and the cochaperone complex prefoldin. Our data strongly support the notion that the prefoldin complex acts to help pVHL folding. The nature of the subunits found in association with pVHL in HEK293 cells supports the idea that it is the “canonical” prefoldin complex, composed of PFDN1-6, which is involved in pVHL folding and not the “PFD-like” complex for which specific subunits like URI were not present in our BioID dataset. In yeast, prefoldin protects pVHL aggregation likely due to hydrophobic residue exposure in the absence of *bona fide* binding partners in the cytosol. Importantly, if aggregates form due to a high level of pVHL expression in yeast, these structures are only transient and ultimately are targeted by intracellular protein degradation mechanisms. The prefoldin subunits may exert complex-mediated chaperone as well as other individual functions in cells. The absence of any of the prefoldin subunits promotes the same aggregation phenotype, strongly supporting a role of the whole prefoldin complex. A similar protective role of the whole prefoldin complex against aggregation and degradation of several misfolded model proteins has been recently demonstrated for all budding yeast GIM mutants [51]. This is further supported by the phenotypic rescue with the full length but not the truncated version of human PFDN3. In the absence of N and C-terminal parts, PFDN3 exhibits reduced complex-mediated function as shown for tubulin or actin [44]. The fact that human PFDN3 fully restores VHL-dependent growth reduction and VHL solubility of the fission yeast *pfd3*Δ mutant emphasizes the conservation of this folding function in eukaryotes. Consistently, we isolated all six subunits of the prefoldin complex with the BioID screen. Prefoldin binds to and transfer unfolded polypeptides to the downstream chaperone complex TRiC/CCT [17,20]. Thus, our work links prefoldin binding to the subsequent folding steps described earlier [37]. Prefoldin may also participate not only in the initial folding step of neo-synthesized pVHL but also by capturing proteins that are released from the chaperonin TRiC/CCT without having reached the native state or later by directing proteins that are denatured (e.g. due to mutations) back to the folding pathway. Our data in Fig 6 suggest that, for patients with mutated VHL, a higher amount of PFDN3 may reduce the severity of ccRCC, possibly by increasing the steady state levels of the whole prefoldin complex and thereby improving pVHL folding efficiency or stability. This might ultimately result in substantial increase of functional pVHL and might impact on VHL-related diseases.

In 2011, Miyazawa *et al*. reported that knock-down of any prefoldin subunit in the H1299 non-small cell lung carcinoma cell line decreased the protein levels of all other subunits, suggesting a protective role of prefoldin complex formation against degradation [46]. However, in their report, PFDN1 siRNA had a modest effect on PFDN3 levels (reduced to 76±10%). Here, we did not observe a significant decrease of PFDN3 in PFDN1 siRNA treated HeLa cells. This small discrepancy may be attributed to the different cell lines.

We identified that prefoldin complex binds to the junction of exon2 and exon3. When only exon1&2 was expressed, the prefoldin binding was severely diminished, ruling out that the hydrophobic stretch is only located in the end of exon 2 but rather overlap with part of the beginning of exon 3. Our data are however in clear contrast with the previous binding site mapping of Tsuchiya *et al*. who identified the last 57 amino acids by a two hybrid approach [29]. With different constructs, we confirmed that the interaction between pVHL and several prefoldin subunits requires both exon 2 and exon 3 (Fig 1A). When comparing alpha tubulin and beta actin prefoldin binding sites, a single consensus binding site could not be defined [24]. This could be explained by the fact that prefoldin subunits which contribute to actin and tubulin binding are different [52]. We did not find an amino acid sequence in pVHL ORF that can match tubulin or actin binding sites either. However, there are two hydrophobic peaks, each on one side of the boundary between exon 2 and exon 3 (Fig 6A). Thus, a possible explanation of the result of Tsuchiya *et al*. [29] is that the two hybrid COOH terminal pVHL fusion protein preserved the second peak in the exon 3 (after aa156) but not the peak of exon 2 (before aa156). We still detected an interaction between exon 3 containing isoforms (e.g. VHL172 see Fig 1E), but it was far less intense compared to the one linking both peaks (Fig 1 and S3 Fig).

When considering a total of 2,154 VHL missense single mutations reported in VHL diseases, this region is mutated in 128 cases [48]. Strikingly, among the mutations found in the aa144-156 sequence, 79 out of 128 cases (61.7%) are corresponding to hydrophobic amino acids that are changed to non-hydrophobic ones, affecting the global hydrophobicity of this region. Three out of four major mutations, accounting for 56 of the 128 cases (43.7%) found in this region (A149S, I151S and I151T), are changes from hydrophobic to non-hydrophobic amino acids. We speculate that the hydrophobic to non-hydrophobic mutations in the aa144-156 may alter the binding of the prefoldin complex and subsequently of the TRiC/CCT complex [32] on pVHL, stimulating degradation of misfolded pVHL. Therefore, these VHL mutations might impact VHL disease severity at least in part by preventing an efficient folding process that normally occurs through binding to prefoldin and/or CCT chaperones.

Importantly, Yang *et al*. reported that one mutation, A149S, promoted pVHL instability. Remarkably, Yang *et al*. were able to show that intervening on pVHL A149S stabilization increased pVHL functionality and yet tumor suppression [12]. A similar stabilization-mediated pVHL functional rescue was also reported for another mutant VHL R167Q [11], suggesting that this approach can be more broadly extended.

It has been shown that prefoldin down-regulation affects microtubule network and expression levels in budding yeast [22,23,53], *A*. *thaliana* [54], *C*. *elegans* [55] and *Drosophila* [31]. Here we show that the function of the prefoldin complex regarding tubulin homeostasis is conserved in fission yeast. We revealed a defect in MT-organization and function for both alpha and beta subunit prefoldin mutants and we confirmed the TBZ sensitivity of all other mutants in addition to *pfd5*Δ [25]. Thus, prefoldin contributes to nuclear centering and chromosome segregation (S6 Fig), albeit symmetry of division and cell survival as consequences did not seem to be affected. In parallel, we showed that MT organization is also altered in mammalian cells with reduced levels of PFDN1, PFDN3 or PFDN5 (S9 Fig). A similar phenotype of disorganized microtubule network was also observed when TRiC/CCT subunit expression is down-regulated [56]. Interestingly, we and others showed that cold or TBZ sensitive phenotypes of yeast prefoldin mutants can be rescued by expression of their human counterparts, supporting a highly conserved function in tubulin regulation [16,25]. However, our work also showed that a truncated version of PFDN3 also rescued growth at 20°C, suggesting that single subunit truncation that did not prevent prefoldin complex formation may not be sufficient to substantially reduce this function.

In addition to study pVHL interacting factors implicated in its folding (prefoldin and TRiC/CCT), our immunoprecipitation experiments identified several proteins that could be more general binding partners of misfolded proteins present in aggregates. Hsp70 ATPase are master regulators of protein folding and degradation. In pVHL immunoprecipitates, seven out of eight of the Hsp70 proteins were recovered, including the main cytosolic Ssa1 and Ssa2 Hsp70. Hsp40/DNAJ co-chaperones stimulates Hsp70 ATPase activity to promote protein folding [57]. Interestingly, six out of 25 of DNAJ domain proteins present in the fission yeast genome were also associated with misfolded pVHL. Two of these DNAJ proteins, Mas5 and Mdj1, have human homologues known to bind to pVHL. Since VHL has no protein partner in fission yeast, the aggregated form is probably a dead-end product. Accordingly, not less than 13 proteasomal subunits both from the base and from the lid structures were found, supporting that pVHL is targeted for degradation. In addition, E3 ligases such as Ltn1 and Hul5 and the Cdc48 ATPase involved in ubiquitin-mediated protein degradation are present in immunoprecipitates. Surprisingly, E3 ligases recently proposed to target pVHL for degradation in budding yeast (UBR1, SAN1, DOA10 and HRD1) were not isolated in our experiments, suggesting that mechanisms of pVHL degradation may differ between the yeast models [58]. The role of other proteins associated with pVHL in fission yeast will be evaluated in the future.

In summary, in addition to key cytoskeletal proteins, we have shown for the first time to our knowledge that the oligohexameric prefoldin cochaperone complex regulates the stability of a tumor suppressor gene product. Recently, the prefoldin complex has been implicated in the folding of the Histone Deacetylase HDAC1 involved in tumorigenesis [59]. Therefore, a broader role of prefoldin complex in pathological processes through the regulation of key proteins folding should now be considered.

## Materials and methods

### *S*. *pombe* strains, media and reagents

*S*. *pombe* strains used in this study were listed in S3 Table. Media and genetic methods were as described [60]. Cells were grown at 30°C. For non-standard growth conditions, cells were grown in medium with 1 mM Bortezomib (ApexBio), 22 μM Geldanamycin (Clinisciences) or DMSO (Sigma). Transformation of plasmids into *S*. *pombe* strains were performed by the lithium acetate procedure. Repression of *nmt1*-derived promoters was achieved by adding 2 μM thiamine in the culture medium. Gene induction from the p*ctr*4^+^ plasmid was carried out by the overnight addition of 50 mM Bathocuproinedisulfonic acid (BCS) in the culture medium [61]. Double mutant strains were obtained by mating single mutant strains on EMM medium without a nitrogen source at 25°C. Subsequent tetrad isolation and dissection were performed using a Singer MSM system tetrad dissector (MSM Singer Instruments, UK).

### *S*. *pombe* protein extract preparation

Cell cultures (50 ml) were harvested by centrifugation at 1700g at 20°C for 2 min, washed in 5 ml ice-cold STOP buffer (10 mM EDTA, 150 mM NaCl, 50 mM NaF, 0.05% NaN_3_). Dried cell pellets were stored at -70°C. Cell pellets were lysed in lysis buffer (30 mM HEPES, pH 8.0, 150 mM NaCl, 1 % glycerol, 1 mM DTT, 0.5% Triton X100, “Cocktail Set IV” Calbiochem protease inhibitor) with 425–600μm glass beads (Sigma) in a Precellys 24 homogenizer (Bertin) at 6100 rpm for 15sec thrice and clarified by centrifugation at 6,000*g* at 4 °C for 5 min. Supernatants were divided in 30-μl aliquots. One aliquot was used as total protein; another aliquot was spun at 16,000*g* at 4 °C for 30 min and designated as the soluble fraction. Pellets were resolubilized by heating in 40μl 1× SDS sample buffer and designed as the insoluble fraction. Ten μl of 4x SDS sample buffer were added to the soluble fraction samples. Equal amounts of each fraction were resolved by SDS–PAGE.

### Human cell lines culture

Human HEK293 and HeLa cells were maintained in Dulbecco’s modified Eagle’s medium and the 786-O cell line in RPMI medium supplemented with 10% fetal bovine serum (FBS) and 1% Antibiotic-Antimycotic solution (Life Technologies) at 37°C and 5% CO_2_.

### Plasmids and strains constructions

#### BioID experiments in mammalian cells

To generate the various VHL-BirA-HA expression constructs, PCR-amplified VHL CDS (full-length or lacking exon1, exon2 or exon3 ORF sequences; Fig 1A) were cloned into the BamHI-linearized pcDNA3.1 MCS-BirA(R118G)-HA vector (Addgene plasmid#36047) using in-fusion cloning (Clontech). In addition, point mutations within the aa144-156 (GQPIFANITLPVY) sequence of pVHL213 were introduced to convert it into a less hydrophobic domain (GQPSTSNSTSPVY). This was achieved by site-directed mutagenesis on using a sequential two-step PCR approach with pcDNA3.1-VHL213-BirA(R118G)-HA as a template and the following mutated oligonucleotides: 5’-AGCCTAGTACTTCCAATAGCACATCGCCAGT-3’ (forward) and 5’-ACTGGCGATGTGCTATTGGAAGTACTAGGCT-3’ (reverse). The resulting cDNA encoding the full-length VHL cDNA with the desired mutations was cloned into the BamHI-linearized pcDNA3.1 MCS-BirA(R118G)-HA vector using in-fusion cloning. All constructs were confirmed by Sanger sequencing.

#### Pfd1 gene deletion in yeast

To delete *pfd1* gene, the whole open reading frame was replaced with the *KanMX6* gene by PCR-based gene targeting as described previously [62]. Stable transformants were selected and screened by PCR for the appropriate gene replacement (*http*:*//www*.*bahlerlab*.*info/home/*).

#### Expression of human Elongin B and Elongin C in yeast

Carboxy-terminal HA_2_-His_6_*-*tagged human EloB construct (pctr4^+^- EloB-HA_2_-HIS_6_) was generated by subcloning a cDNA encoding human EloB into SpeI/NotI-digested pctr4^+^-X- HA_2_-HIS_6_ plasmid [61]. This cDNA was obtained by PCR amplification from pST39-HisTrxNVHL-ElonginB-ElonginC [63] with specific oligonucleotides containing SpeI and NotI restriction sites and digested with SpeI and NotI enzymes.

NH2-terminal *myc*-tagged human EloC construct (pctr4^+^-Myc-EloC) was generated by subcloning a cDNA encoding Myc-EloC into SpeI/EagI-digested pctr4^+^-X_*EagI*_ plasmid [61]. This cDNA was obtained by PCR amplification from pESC-LEU-ELONGIN B/C [64] with specific oligonucleotides containing SpeI and EagI restriction sites and digested with SpeI and EagI enzymes.

#### Expression of full-length or truncated human PFDN3/VBP1 in yeast

Expression of human PFDN3 (VBP1) in yeast was allowed by transforming *S*. *pombe* with the pSM495 plasmid (cDNA encoding AA32-197 of PFDN3 isoform 1 [NP_003363.1] inserted between BamHI and XhoI restriction sites of p415ADH) [16]. Besides, truncated version of human PFDN3 (AA63-165; *i*.*e*. devoid of its NH2- and COOH terminal parts) was PCR-amplified from pSM495 plasmid and subcloned into the BamHI/XhoI-digested p415ADH plasmid using in-fusion cloning (Clontech).

#### Insertion cassettes cloning and integration in yeast genome

GFP and GFP-pVHL213 ORF cDNAs were inserted into the SalI-linearized pINT5 plasmid using in-fusion cloning (Clontech). These constructs were released by NotI enzyme digestion and integrated by homologous recombination after transformation of a *ura4-D18 h*^*-*^ strain. Stable *ura4+ leu1-* colonies were selected on EMM plates supplemented with leucine. All inserts were confirmed by Sanger sequencing.

### Proximity biotinylation (BioID) in mammalian cells and affinity capture of biotinylated proteins

HEK293, HeLa, or 786-O cells plated in 15-cm dishes were transfected with empty pCDNA3.1-BirA(R118G)-HA or pCDNA3.1-VHL-BirA(R118G)-HA plasmids using JetPrime (Ozyme) or HeLaFect (OZ Biosciences) reagents as recommended by the manufacturers and further cultured in media supplemented with 50 μm biotin from 4 to 24 hours following transfection. They were washed 3 times in PBS, harvested and stored at -70°C until use. Pelleted cells were lysed in RIPA buffer (50 mM Tris-HCl, pH 7.6, 150 mM NaCl, 1 mM EDTA, 1 mM EGTA, 0.1% SDS, 1% Triton X100 and protease inhibitor cocktail (Thermo Fisher Scientific)), sonicated and centrifuged at 16,000 *g* for 15 min at 4°C. Total proteins in supernatants were quantified by Bio-Rad assay and equal amounts of total proteins in each experiment were used for streptavidin capture. Before the affinity chromatography, aliquots of total proteins were saved for each condition (“input” fractions). Streptavidin-Sepharose Beads (60 μl, BioVision) were washed 3 times with lysis buffer and incubated with total protein lysates for 3h30 at 4°C on a tube rotator. Protein-bound beads were then washed three times with RIPA buffer and three times with 50 mM ammonium bicarbonate pH 7.8. Elution of biotinylated proteins (“Bound” fraction) was achieved by adding 50 μl 2xLaemmli loading buffer to the beads and eluted proteins were denatured by heating at 95°C for 5 min prior to western blot analysis.

### Prefoldins knock-down in mammalian cells

Small interfering RNA (siRNA) transfections were performed with JetPrime (Ozyme) according to the manufacturer’s instructions. Cells were treated with 40 nM siRNA duplexes against human Pfd1 and/or Pfd3 (sc-40869 and sc-40873, respectively; Santa Cruz Biotechnology), or a MISSION siRNA Universal Negative Control duplex (Sic001; Sigma) for 48 hr. They were then washed twice in PBS, harvested and stored at -70°C until further analysis (RT-qPCR or western blotting) or fixed for immunofluorescence. Knock-down efficiency was assessed by RT-qPCR and/or by western blotting.

### RNA extraction and RT-qPCR analysis

Total RNA was extracted from cells using the Nucleospin RNA reagent kit (Macherey-Nagel). cDNAs were synthesized from 2 μg total RNA using 500 ng random hexamer primers and 200 U M-MLV reverse transcriptase (Promega) per reaction as recommended by the manufacturer. Primers used for quantitative PCR (qPCR) were as follows: 5’-ATCACCCTGAGCCCCATTTG-3’ (forward) and 5’-TCCCATCTTCCCTCTCCTGG-3’ (reverse) for hPFDN1; 5’-AAGGACAGTTGTGGCAAAGG-3’ (forward) and 5’-TCTCATTCCCAGGCTGTTTC-3’ (reverse) for hPFDN3; 5’-GACACACGATGGGCTTCTG -3’ (forward) and 5’-GCATCCACAGCTACCGAGTGTA -3’ (forward) for hVHL variant 1 (NM_000551) and variant 2 (NM_198156) respectively, with the same reverse primer 5’-TGACGATGTCCAGTCTCCTG-3’ for both VHL variants; 5’-AATGACCCCTTCATTGACCTC-3’ (forward) and 5’-TTCCATTGATGACAAGCTTCC-3’ (reverse) for hGAPDH as control. qPCR was performed using power SYBR Green PCR Master mix (Life Technologies) as previously described [4].

### Immunoprecipitation

#### From yeast cells

Yeast cell cultures (500ml) were harvested by centrifugation at 1700*g* at 20°C for 5 min, washed in 50ml ice-cold PBS buffer. Dried cell pellets were stored at -70°C. Cell pellets were lysed in 10ml lysis buffer (20mM Tris HCl pH7.5, 150mM NaCl, 2mM EDTA, 0.5% Nonidet P40, Protease Inhibitor Cocktail Tablet Roche) with 425–600μm glass beads (Sigma) in the Precellys 24 homogenizer (Bertin) at 6100 rpm for 30sec six-fold and clarified by centrifugation at 6,000*g* at 4 °C for 10 min. One aliquot of supernatant was used as input proteins and 9ml of remaining (29mg of proteins) incubated with 100μl GFP-Trap MA beads (Chromotek) 90min at 4°C under rotation. The beads were washed thrice with lysis buffer, thrice with buffer without Nonidet P40 and finally resuspended in 50 mM NH_4_HCO_3_ pH8.0.

#### From mammalian cells

Human HeLa cells pellets (from transfected cells in 15-cm culture dishes) were lysed in 200μl lysis buffer (10mM Tris HCl pH7.5, 150mM NaCl, 0.5mM EDTA, 0.5% Nonidet P40, Protease Inhibitor Cocktail Tablet Roche) 30 min in ice, regularly vortexed, centrifuged 10 min at 11,000*g*. Total proteins in supernatants were quantified by Bio-Rad protein assay and equal amounts of total proteins in each experiment, diluted in buffer (10mM Tris HCl pH7.5,150mM NaCl, 0.5mM EDTA) were used for immunoprecipitation with 25μl GFP-Trap MA beads (Chromotek). After 90 min at 4°C under rotation the beads were washed 4 times with dilution buffer and finally resuspended in 2xLaemmli loading buffer.

### GST-TUBE (Tandem-repeated Ubiquitin-Binding Entities) pull-down assay

#### Immobilization of GST-TUBE on glutathione-agarose sepharose beads

Glutathione-S-transferase (GST) fusion protein was purified as follows: Briefly, pGEX-6P1 plasmid containing repeated uba1 domains of human HR23A [65] was transformed into BL21 E. coli cells (New England Biolabs) and colonies were obtained by selective growth in solid Luria Bertani medium (LB) plus ampicillin at 37°C.

Expression of GST-fusion protein was induced with 0.5 mM IPTG for 2 h at 37°C. The bacteria were centrifuged at 3,000*g* for 15 min at 4°C and washed in ice-cold PBS. After centrifugation, the pelleted bacteria were lysed during a 30-min incubation in 25 ml ice-cold PBS containing 1 mg/ml lysozyme and 1 mM AEBSF. The lysate was then sonicated, successively syringed though a 18-, 23-, and 25-gauge needles and centrifuged at 10,000*g* for 30 min to remove debris. The supernatant (that contains soluble GST-TUBE; 800 μl) was 0.45-μm filtered and incubated with equilibrated glutathione-agarose beads (Clinisciences; 70 μl) for 30 min at room temperature, with gentle agitation and the beads were rinsed 3 times with ice-cold PBS.

#### Preparation of cell extracts

Parental 786–0 cells, pVHL213- or pVHL172-expressing 786–0 cells were first incubated at 37°C for 12 hours in RPMI containing antibiotics, 10% FBS and 100nM bortezomib, harvested and lysed with chilled TUBEs lysis buffer (50 mM sodium fluoride, 5 mM tetra-sodium pyrophosphate, 10 mM β-glycerophosphate, 1% Igepal CA-630, 2 mM EDTA, 20 mM Na_2_HPO_4_, 20 mM NaH_2_PO_4_, 5 mM AEBSF and 1x complete protease inhibitor cocktail; Roche, France) containing 100 mM 2-chloroacetamide to minimize DUB activity. Lysates were sonicated, centrifuged at 16,000*g* for 15 min at 4°C. Yeast cell extracts were prepared as indicated above from cells cultured overnight in a medium with 1mM bortezomib. Protein concentration in resulting supernatants was determined using the Bio-Rad protein assay reagents.

#### GST pulldown

Cleared lysates (500–750 μg total proteins) were subjected to GST pull-down by addition to GST-TUBE previously loaded on glutathione-agarose beads and incubation at 4°C for 2 hours under end-to-end rotation. After washing the beads with chilled lysis buffer for 4 times, the captured ubiquitinated proteins (bound fraction) were eluted upon addition of 80 μl 1.5x Laemmli buffer, heat-denatured at 95°C for 5 min in parallel to total protein samples (Input) and subjected to SDS-PAGE followed by Coomassie blue staining (Bio Safe Coomassie G250 stain, Bio-Rad) or immunoblotting.

### On-beads protein digestion

Protein extracts containing VHL-interacting proteins were resuspended in 100 μL of 75 mM ammonium bicarbonate buffer pH 8.0. Proteins were reduced with 10 mM DTT for 20 min and alkylated in the dark with 50 mM iodoacetamide. Incubations were performed at room temperature on a tube rotator to ensure homogeneous suspension of the beads. Two micrograms of a Trypsin/Lys-C protease mix (Promega Corporation, USA) were added to samples and incubated overnight at 37°C on a tube rotator. Samples were supplemented with 2 μg of SOLu-Trypsin (Millipore-Sigma, USA) and incubated for an additional 3 hrs at 37°C to promote the completeness of the digestion process. The digested products were acidified with trifluoroacetic acid (TFA) and the peptides were isolated and washed on C18 tips (Thermo Fisher Scientific, USA) according to the manufacturer's instructions. Finally, the purified peptides were dried by vacuum centrifugation and stored at −80°C.

### Mass spectrometry

Peptide samples were analyzed using LC-MS/MS on two instruments. Approximately half of the sample was analyzed on a Q Exactive OrbiTrap mass spectrometer (Thermo Fisher Scientific, USA) while the other half was analyzed on a timsTOF Pro mass spectrometer (Bruker Daltonics, USA). For the analysis on the Q Exactive OrbiTrap instrument, trypsin-digested peptides were separated using a Dionex Ultimate 3000 nanoHPLC system. Ten μl of sample (a total of 2 μg) in 1% (vol/vol) formic acid were loaded with a constant flow of 4 μl/min onto an Acclaim PepMap100 C18 column (0.3 mm id x 5 mm, Dionex Corporation). After trap enrichment, peptides were eluted onto an EasySpray PepMap C18 nano column (75 μm x 50 cm, Dionex Corporation) with a linear gradient of 5–35% solvent B (90% acetonitrile with 0.1% formic acid) over 240 minutes with a constant flow of 200 nl/min. The HPLC system was coupled to the mass spectrometer via an EasySpray source. The spray voltage was set to 2.0 kV and the temperature of the column set to 40°C. Full scan MS survey spectra (*m/z* 350–1600) in profile mode were acquired in the Orbitrap with a resolution of 70,000 after accumulation of 1,000,000 ions. The ten most intense peptide ions from the preview scan in the Orbitrap were fragmented by collision-induced dissociation (normalized collision energy 35% and resolution of 17,500) after the accumulation of 50,000 ions. Maximal filling times were 250 ms for the full scans and 60 ms for the MS/MS scans. Precursor ion charge state screening was enabled and all unassigned charge states as well as singly, 7 and 8 charged species were rejected. The dynamic exclusion list was restricted to a maximum of 500 entries with a maximum retention period of 40 seconds and a relative mass window of 10 ppm. The lock mass option was enabled for survey scans to improve mass accuracy. Data were acquired using the Xcalibur software.

For the analysis on the timsTOF Pro mass spectrometer, samples were injected into an HPLC (nanoElute, Bruker Daltonics, USA) and loaded onto a trap column with a constant flow of 4 μl/min (Acclaim PepMap100 C18 column, 0.3 mm id x 5 mm, Dionex Corporation) and eluted onto an analytical C18 Column (1.9 μm beads size, 75 μm x 25 cm, PepSep). Peptides were eluted over a 2 hrs gradient of ACN (5–40%) in 0.1% FA at 400 nL/min while being injected into the mass spectrometer equipped with a Captive Spray nano electrospray source (Bruker Daltonics, USA). Data was acquired using data-dependent auto-MS/MS with a 100–1700 *m/z* mass range, with PASEF enabled with a number of PASEF scans set at 10 (1.27 seconds duty cycle) and a dynamic exclusion of 0.4 minute, *m/z* dependent isolation window and collision energy of 42.0 eV. The target intensity was set to 20,000, with an intensity threshold of 2,500.

### Analysis of mass spectrometry data

Spectral files generated on the Q Exactive OrbiTrap and timsTOF Pro mass spectrometers were searched using Byonic version 3.3.9 (Protein Metrics, USA) against the *Schizosaccharomyces pombe* reference proteome (Uniprot, 5141 protein entries) with a static modification of carbamidomethyl (+57.0215 Da on Cys) and the following variable modifications: oxidation of methionine (+15.9949 Da), deamidation of glutamine and asparagine (+0.9840 Da) and the formation of pyro-Glu from N-terminal glutamate and glutamine residues (-18.0105 Da for N-Term Glu and -17.0265 Da for N-term Gln). Semi-specific trypsin cleavage was specified and a maximum of two missed cleavage was allowed. The mass tolerance was set to 7 ppm for the precursor ions and 20 ppm for the fragment ions for the analysis with the Q Exactive OrbiTrap mass spectrometer. The mass tolerance was set to 70 ppm for the precursor ions and 35 ppm for the fragment ions for the analysis with the timsTOF Pro. A false discovery rate (FDR) of <1% was estimated using concatenated forward–reverse database search at the peptide-spectrum match (PSM) level. Byonic output files corresponding to each sample were merged with Batcher version 3.3–369 (Protein Metrics, USA). Decoys protein IDs and common contaminants were filtered out of the final dataset (*e*.*g*., trypsin, serum albumin, human keratins, etc). Only proteins with an identification p-value ≤ 0.001 (i.e. a Log base 10 of the protein p-value ≥ 3) were reported.

### Analysis of gene ontology enrichment

Gene names corresponding to proteins identified by LC-MS/MS in GFP (control) and VHL-GFP affinity-purification extracts (refer to S1 Table) were used to compare results with Venn diagram (https://bioinfogp.cnb.csic.es/tools/venny/index.html). Gene symbols unique to VHL-GFP extracts were submitted as a gene list to DAVID online functional annotation tool [66,67] and all enriched gene ontology (GO) annotations were retrieved using the GO FAT database. The DAVID analysis returned a typical list of enriched GO categories (broad GO terms as specified with the GO FAT filter). The most significant terms associated with each GO categories (i.e. biological process, cellular component and molecular function) were selected.

### Western blotting

Proteins were resolved by SDS–PAGE and transferred onto nitrocellulose membranes. Unless otherwise specified, membranes were blocked with 5% non-fat dry milk in TBS/0.1% Tween 20 (TBST/milk) and incubated overnight at 4°C with the following primary antibodies diluted in TBST/milk: rabbit monoclonal α-cullin 2 (Invitrogen; 1:500), rabbit polyclonal α-Elongin C (BioLegend; 1:1000), rabbit polyclonal α-ERK_1/2_ (Santa Cruz Biotechnology; 1:3000), rabbit polyclonal GAPDH (Cusabio; 1:3000), mouse monoclonal α-GFP (Roche; 1:4000), rat monoclonal α-HA (Roche; 1:4000), rabbit monoclonal p21^WAF1/CIP1^ (Cell Signaling Technology; 1: 1000), rabbit polyclonal α-Prefoldins 1, 2, 4 and 5 (Abclonal; 1:1000–1:3000), mouse monoclonal α-prefoldin3/VBP1 (Santa Cruz Biotechnology; 1:250), mouse monoclonal α-PSTAIR (Sigma; 1:5000), mouse monoclonal α-ubiquitin (P4D1; Santa Cruz Biotechnology; 1:1000), mouse monoclonal α-HSP70 (Abcam; 1:1000) and mouse monoclonal α-VHL [68] (1:1000). Horseradish peroxydase-conjugated secondary antibodies used were purchased from Jackson Immunoresearch: rabbit α-mouse IgG (1:25,000), goat α-rabbit IgG (1:30,000) and goat α-rat IgG (1:10,000). Immunocomplexes were detected with ECL Select substrate (GE Healthcare) on the Amersham gel imager 680 (GE Healthcare). Quantification of Western blots by densitometry was performed using the ImageQuant software (GE Healthcare). GADPH or Cdc2 (with the PSTAIR antibody) were used as loading controls to normalize the quantification.

### Microscopy techniques

Live and fixed yeast cells were captured using Leica DMRXA with the Metamorph software or with a Deltavision microscope with deconvolution. Aggregate containing cells were scored with Metamorph or ImageJ Cell Counter plugin (at least n = 50 cells per condition). Nuclei were stained with 200 ng/ml DAPI (Sigma). HeLa cells were cultivated on coverslips and fixed with 4% paraformaldehyde for 10 min, permeabilized with 0.2% Triton X-100 in PBS and stained after blocking with 1% BSA in PBS. Primary anti-tubulin antibodies (dilution 1:100), were incubated onto the cells for 2 h at room temperature. After washing, the secondary antibodies directed against mouse IgG were incubated for 1h at room temperature. The nuclei were stained with DAPI/Antifade (Q-BIOgene MP Biomedicals, Illkirch, France). Samples were examined using a Deltavision microscope.

### Proximity ligation assay

The 786–0 (VHL-/-) or 786-O-pVHL213 (stably expressing Flag-HA-pVHL213) cells were cultivated on coverslips for 24 hours. The cells were then fixed with 4% PFA for 10 min at room temperature permeabilized with 0.1% Triton X-100 in PBS and blocked in the blocking buffer for 1h at 37°C (DuoLink -Sigma). The cells were washed and processed for labelling using Duolink PLA fluorescence Protocol from Sigma. After blocking, the cells were incubated over night at 4°C with the following antibodies: anti HA (Roche) dil:1:600 and polyclonal anti VBP1/PFDN3 (Proteintech) dil:1:600. After washing, in buffer A (DuoLink-Sigma) the cells were incubated in the presence of anti-Rabbit IgG PLUS and anti-mouse IgG (H+L)Plus. The cells were washed in buffer B (DuoLink-Sigma), the amplification signal was realized with the polymerase in the presence of fluorescently labeled complementary oligonucleotides probes and the signals were observed with a Leica DMI 6000 CS microscope.

### Secondary structure prediction

For prediction of prefoldin subunit secondary structures, we used the PSIPRED software (http://bioinf.cs.ucl.ac.uk/psipred/)

### Statistical analyses

All statistical analyses were done with R studio. The error bars represents ±S.D (unless otherwise specified) from experimental independent replicates of at least n = 3. Statistical analyses were conducted with Student t-test, Kruskal-Wallis or Mann-Whitney tests.

## Supporting information

S1 Fig PFDN1 and PFDN3 proteins are recovered in streptavidin pulldowns after expression of pVHL-BirA fusion proteins but not BirA in 786-O cells. Western blot analysis of PFDN1 and PFDN3 and BirA fusion proteins in total protein extracts before (Input) and in fractions eluted out from the Streptavidin affinity-chromatography column (Bound). Ctl depicts untransfected control cells. GAPDH was used as a negative control.
(PDF)

S2 Fig Co-localization of PFDN1 and PFDN3 with pVHL213 using Proximity Ligation Assay. 786-O cells (as a negative control, VHL-/- cells) and 786-O-pVHL213 cells (cells stably expressing Flag-HA-VHL213) have been processed for a Proximity Ligation Assay (PLA) using anti-PFDN1, anti-PFDN3 and anti-HA antibodies alone or in combination as indicated on the right. Representative confocal microscopy images generated from PLA are shown: PLA signals in reversed fluorescence (left) and superposition of DAPI (blue) and PLA (red) signals (right).
(PDF)

S3 Fig The VHL213mut variant, lacking the aa144-156 hydrophobic peak, binds poorly to the prefoldin complex. A) Scheme depicting the impact of site-directed mutagenesis on the hydrophobicity of the aa144-156 region of pVHL213. Hydrophobicity of the aa144-156 region is indicated as a Kyte-Doolittle plot for wild type VHL213 aa144-156 wt (GQPIFANITLPVY, black line) and the mutated VHL213mut aa144-156 mut (GQPSTSNSTSPVY, dashed line). B) Western blot analysis of PFDN1, PFDN3, PFDN5 and BirA (HA) fusion proteins in total protein extracts from HEK293 cells (Input) and of fractions eluted from the Streptavidin-sepharose beads (Bound). Cullin 2 (CUL2) was used as positive control whereas p44/42 ERK was used as negative control. Ctl corresponds to untransfected control cells. A long exposure for PFDN5 is shown on the right. C) Quantification of the biotinylated prefoldin / pVHL expression levels for VHL213wt-, VHL213mut- and VHL172-BirA fusion proteins. Histograms represent the mean ratios of biotinylated PFDN1, PFDN3, PFDN5 and CUL2 proteins (VHL binding partner) on total pVHL expression. For each analyzed protein, the ratio was set as 100% in full-length VHL213wt-expressing cells. Mean±s.d. from three independent experiments, n.s not significant; **, p-value<0.01; ***, p-value<0.001; VHL213 wt *vs* VHL213 mut and VHL172 for each VHL binding partner, Mann-Whitney test)
(PDF)

S4 Fig Proteomics analysis of VHL-interacting proteins. A) Venn diagram comparison of proteins identified by LC-MS/MS in GFP (control) and VHL-GFP affinity-purification extracts. B) Most significant over-represented functional categories classed by gene ontology (GO) for VHL-specific interactors.
(PDF)

S5 Fig Prefoldin subunits are structurally conserved in evolution. The NH_2_- and COOH-terminal regions of prefoldin subunits are formed by α-helices (pink) that are connected by β-hairpin linkers. Each β-hairpin linker consists of four short β-strands (yellow) for α prefoldin subunits (PFDN3, PFDN5, Pfd3, Pfd5) and usually one or two short β-strands for β prefoldin subunits (PFDN2, PFDN6, Pfd2, Pfd4, Pfd6). No short β-strands were predicted by PSIPRED between α-helices for prefoldin subunits Hs PFDN1, Hs PFDN4 and Sp Pfd1. Hs: *Homo sapiens*, Sp: *Schizosaccharomyces pombe*.
(PDF)

S6 Fig Fission yeast prefoldin subunit mutants show microtubule network deficient phenotypes. A) Serial dilutions (1:5) of wild-type and *pfd* deletion mutants were spotted on YES plates (YES 30°C) or YES plates containing 7.5 mM microtubule-depolymerizing Thiabendazole (YES+TBZ 30°C) at 30°C (2 days) or on YES plates at 20°C for 4 days (YES 20°C). B) Cellular phenotypes of nuclear positions or mitotic defects of fission yeast prefoldin mutants. Percentage of cells showing C) an asymmetric nucleus or D) mitotic defects in WT and prefoldin mutants at 20°C and 30°C (mean±s.d. from three independent experiments). E) Microtubule network organization in WT, *pfd1*Δ and *pfd3*Δ cells expressing GFP-tagged α-tubulin (Atb2). Bar: 5 μm. F) Histogram reporting the number of MT bundles observed per cell in WT, *pfd1*Δ and *pfd3*Δ strains. (n.s., non significant; ***, p<0.001). G) Western blot analysis of GFP-Atb2 expression in WT, *pfd1*Δ and *pfd3*Δ strains. Cdc2 was used as a loading control.
(PDF)

S7 Fig Fission yeast *pfd* mutants do not exhibit a general protein aggregation phenotype. A and B) Steady-state and heat-induced Hsp70 expression levels are similar in wild type and prefoldin mutant strains. A) Western blot analysis of Hsp70 expression levels in wild type (WT) and prefoldin mutants. Cells were grown to exponential growth phase at 30°C to prepare whole cell protein extracts. At the bottom of the Hsp70 gel are indicated the mean relative amounts of Hsp70 in the different strains (WT was set to 1; mean of 3 experiments, no significant difference, Kruskal-Wallis test). B) Western blot analysis of Hsp70 expression levels in wild type (WT) and *pfd1*Δ and *pfd3*Δ mutants at 30°C and after a 2-hour heat shock at 37°C. Cells were grown to exponential growth phase at 30°C and cultures were split in two samples. One was left at 30°C (left panel) and the other half was incubated for 2h at 37°C (right panel). Cdc2 was used as a loading control. C) Wild type and prefoldin mutant strains show similar thermotolerance. The growth phenotypes of the wild type (WT) and *pfd* mutant strains were compared to the thermotolerance-deficient *hsp104*Δ mutant and to the *mas5*Δ mutant which exhibits a high Hsf1 constitutive activity. Wild type and mutant cells were grown to mid-log phase and were either spotted on YES plates at 25°C for 3 days (left panel) or shifted to 50°C for 15 minutes before being spotted at 25°C (middle panel). An additional sample was first shifted to 37°C for 45 minutes before being exposed to 50°C (right panel). D) Wild type and *pfd3*Δ cells exhibit similar Hsp104-GFP distributions. Reversed images (upper panels) and quantification of Hsp104-GFP dot containing cells (bottom panel) of wild-type (WT) or *pfd3*Δ cells at 20°C or 30°C. E) Wild type and prefoldin mutant strains show similar sensitivity to the proline analog azetidin-2-carboxylic acid (AZC) which promotes general protein misfolding. The growth phenotypes of the indicated wild type (WT) and *pfd* mutant strains were compared to the AZC-sensitive AZC acetyltransferase deletion mutant *ppr1*Δ strain by serial dilutions on EMM and 0.75 mg/ml AZC-containing EMM plates at 30°C for 6 days. Note that *ade6*^-^ mutant strains (*pfd2*Δ, *pfd3*Δ, *pfd4*Δ and *pfd5*Δ) were darker on AZC-containing plates due to a red staining. This indicates that AZC interferes with adenine uptake in *ade6*^-^ mutant even though adenine was not in limiting amounts in the culture medium. F) Analysis of total ubiquitinated protein levels in wild type and prefoldin mutant strains. Wild type (WT), *pfd1*Δ and *pfd3*Δ strains were grown to mid-log phase. Total protein extracts were prepared and incubated with GST-TUBE-loaded glutathione-agarose beads (see Materials and Methods). Upper panel: Coomassie blue staining of total protein extracts (Input, left panel) from WT, *pfd1*Δ and *pfd3*Δ cultures and fractions after elution from glutathione-agarose beads (Bound, right panel). Lower panel: Western blot analysis of total ubiquitinated proteins in the bound fractions revealed by an anti-P4D1 antibody (ubiquitin). At the bottom of the gel are indicated the relative amounts of ubiquitinated proteins (WT was to 1; no significant difference was observed between the samples, Mann-Withney test). MW (upper panel), Molecular weight markers as in lower panel; « no » indicated that no input protein extract was incubated with GST-TUBE-loaded beads; The last lane in A and B gels corresponded to elution fraction from unloaded glutathione-agarose beads incubated with a *pfd3*Δ protein extract (control). G) Expression of the soluble GFP or the low aggregation propensity exon2-deleted GFP-tagged pVHL172 isoform in wild-type and *pfd* deletion mutants: reversed fluorescent images of cells in the presence of DMSO or BZ. Bars: 5 μm.
(PDF)

S8 Fig Microtubule network deficiency does not impact pVHL213 aggregation pattern. A) The effect of two concentrations of the MT-depolymerizing drug, thiabendazole (TBZ), on the MT network organization was assayed by imaging GFP-Atb2 (alpha-tubulin) in fission yeast cells: deconvolved GFP fluorescence (upper panels) and phase contrast (lower panels). B and C) The impact of two concentrations of TBZ and of the MT-deficient *tea2*Δ mutant on pVHL213 aggregation was monitored: B) Reversed deconvolved fluorescent images of GFP-VHL213 expressing cells and C) Histogram representing the percentage of cells with low (<10 aggregates/cell), medium (20–30 aggregates/cells) or high (>30 aggregates/cell) pVHL213 aggregation patterns (mean of 3 independent experiments; DMSO, n = 802; 50 μg/ml TBZ, n = 693; 100 μg/ml TBZ, n = 635; *tea2*Δ, n = 175). Statistical tests showed no significant difference (n.s.) between the samples. Bars: 5 μm.
(PDF)

S9 Fig Silencing of *PFDN* genes in HeLa cells affects microtubule organization. Representative immunofluorescence images of the microtubule network of untreated (no) HeLa cells or siRNA treated cells with control SiRNA (siCtl) or siRNA targeting either *PFDN1*, *PFDN3* or *PFDN5* genes. Bar: 10 μm.
(PDF)

S10 Fig Effect of downregulation of PFDN1, PFDN3 and PFDN1+PFDN3 on pVHL172 stability in human cells. A) Western blot analysis of pVHL172 expression after siRNA-driven knock-down of PFDN1, PFDN3 or PFDN1+PFDN3 in HeLa cells. B) Histogram representing the pVHL172 levels in PFDN siRNA experiments (mean±s.e.m from at least three independent experiments). “Si Ctl” represents cells transfected with mock siRNA. *, p-value<0.05; **, p-value<0.01. Mann-Whitney test.
(PDF)

S11 Fig PFDN gene silencing does not down-regulate the VHL gene transcription. RT-qPCR analysis of PFDN1, PFDN3, full-length VHL213 and exon-2 deleted VHL172 mRNA expression in HeLa cells treated with PFDN1, PFDN3, PFDN1+PFDN3 siRNA (Si) or control siRNA (Si Ctl) or untreated cells (no; tranfectant only). The GAPDH gene expression was used for normalization (n = 4). The mRNA levels in untreated cells were set to 1 for each condition. No significant differences were observed between the samples except when indicated * (p<0.05); Mann-Whitney test.
(PDF)

S12 Fig The PFDN1 and PFDN3 expression levels are independent of the pVHL status in human cell lines. A) Western blot analysis of PFDN1 and PFDN3 in protein extracts from the 786-O parental cell line (VHL-/-), the 786-O-pVHL213 and 786-O-pVHL172 cell lines [4]. HIF2α, a target of the pVHL213 VBC E3 ligase complex, was used as a positive control for E3 ligase activity whereas GAPDH and β-tubulin were used as loading controls. B) Quantification of the expression levels of PFDN1, PFDN3 and HIF2α. Mean±s.d. from three independent experiments, no significant difference for samples except PFDN3 in 786-O-pVHL172 and HIF2α in 786-O-pVHL213 (*, p-value<0.1; Mann-Whitney test). C) Quantification of PFDN1 and PFDN3 expression levels after a Western blot analysis in total protein extracts from HEK293 cells transiently transfected with wild type VHL213 (VHL213 wt), mutated VHL213 deficient for prefoldin binding (VHL213 mut) or VHL172. The levels of PFDN1 and PFDN3 were set as 100% in the control untransfected cells. No significant difference were observed between all samples (Mann-Whitney test)
(PDF)

S13 Fig The PFDN1 and PFDN3 subunits are very unlikely targeted by pVHL213 for ubiquitination. Western blot analyses of total protein extracts (Input) and of pulled-down (bound) fractions eluted from glutathione-agarose-beads loaded or not with GST-TUBE. The cells lines were 786-O (VHL-/-) and 786-O cells expressing either pVHL213 (786-O VHL213) or pVHL172 (786-O VHL172). As controls (Ctl) in pulled-down fractions, no protein extract was loaded on a GST-TUBE-loaded beads (first lane) or only on unloaded glutathione-agarose beads (last lane). Another negative control was a 786-O VHL213 extract incubated with unloaded gluthathione-agarose beads (penultimate lane). The following proteins were detected in Western blots using specific antibodies: A) total ubiquitinated proteins (using P4D1 antibody), B) HIF2α, C) GAPDH (negative control), D) pVHL isoforms, E) PFDN1, F) PFDN3, G) p21^CIP1^.
(PDF)

S1 Table Mass spectrometry analysis of proteins isolated in GFP immunoprecipitates from *leu1*::*GFP-pVHL213* and *leu1*::*GFP* control cells. (XLS)

S2 Table Sequence conservation of prefoldin subunits in model eukaryotes. *Schizosaccharomyces pombe* prefoldin subunit encoding genes (*pfd1* to *pfd6*) were named according to their human counterparts (Human homologue). The amino acid identities with prefoldin subunits of different model organisms (budding yeast *Saccharomyces cerevisiae*, the nematode *Caenorhabditis elegans*, the fruit fly *Drosophila melanogaster*, the mouse *Mus musculus* and human *Homo sapiens)* are indicated on the right.
(PDF)

S3 Table *S*. *pombe* strains used in this study. (DOCX)
