# Supplementary figures and images for: The prefoldin complex stabilizes the von Hippel-Lindau protein against aggregation and degradation
Source: PLoS Genet. 2020 Nov 2;16(11):e1009183. doi: 10.1371/journal.pgen.1009183 (PMC7660911; doi:10.1371/journal.pgen.1009183)

786-O

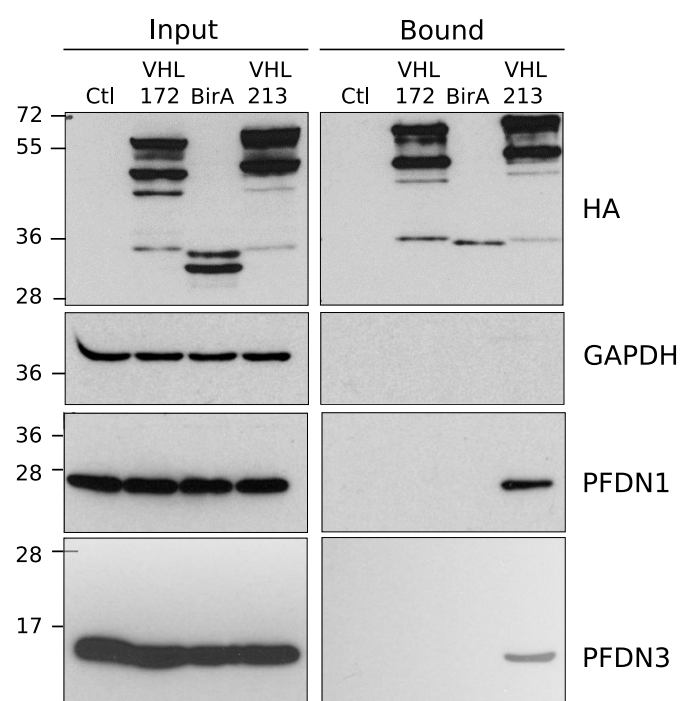

S1 Fig

Supplement: S1 Fig — Western blot analysis of PFDN1 and PFDN3 and BirA fusion proteins in total protein extracts before (Input) and in fractions eluted out from the Streptavidin affinity-chromatography column (Bound). Ctl depicts untransfected control cells. GAPDH was used as a negative control. (PDF) [file pgen.1009183.s001.pdf]

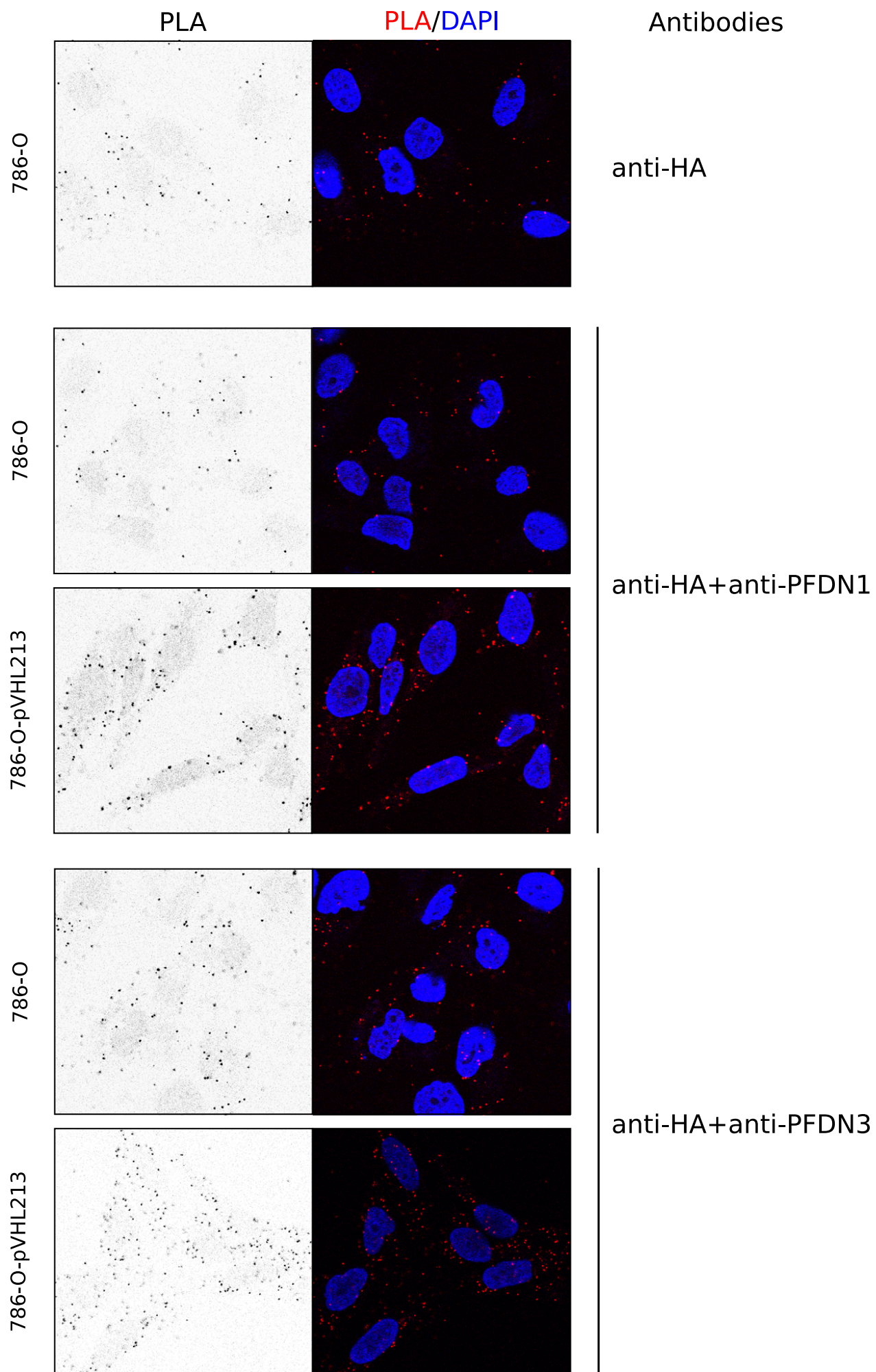

S2 Fig

Supplement: S2 Fig — 786-O cells (as a negative control, VHL-/- cells) and 786-O-pVHL213 cells (cells stably expressing Flag-HA-VHL213) have been processed for a Proximity Ligation Assay (PLA) using anti-PFDN1, anti-PFDN3 and anti-HA antibodies alone or in combination as indicated on the right. Representative confocal microscopy images generated from PLA are shown: PLA signals in reversed fluorescence (left) and superposition of DAPI (blue) and PLA (red) signals (right). (PDF) [file pgen.1009183.s002.pdf]

**A**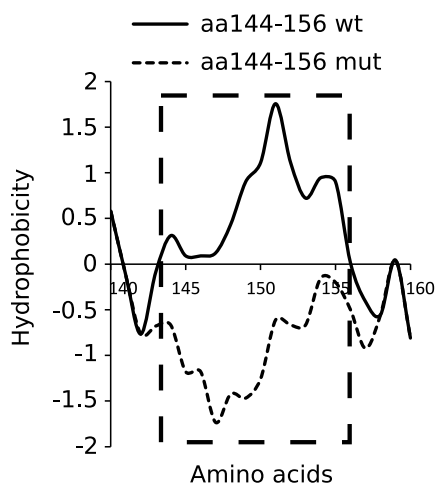**C**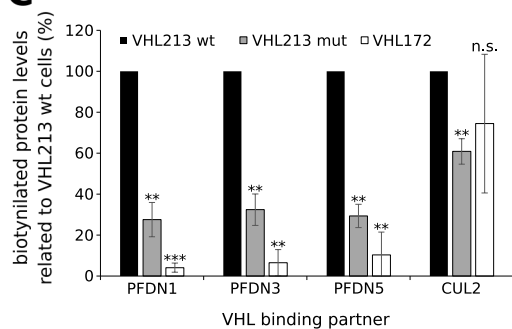**B**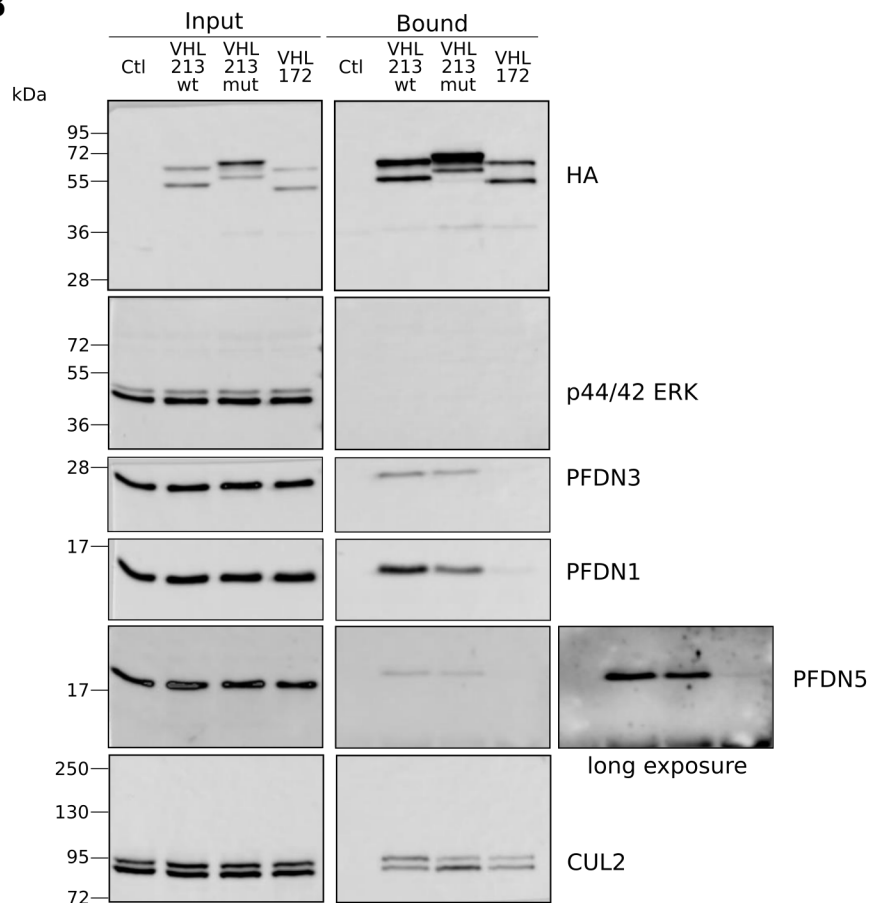

S3 Fig

Supplement: S3 Fig — A) Scheme depicting the impact of site-directed mutagenesis on the hydrophobicity of the aa144-156 region of pVHL213. Hydrophobicity of the aa144-156 region is indicated as a Kyte-Doolittle plot for wild type VHL213 aa144-156 wt (GQPIFANITLPVY, black line) and the mutated VHL213mut aa144-156 mut (GQPSTSNSTSPVY, dashed line). B) Western blot analysis of PFDN1, PFDN3, PFDN5 and BirA (HA) fusion proteins in total protein extracts from HEK293 cells (Input) and of fractions eluted from the Streptavidin-sepharose beads (Bound). Cullin 2 (CUL2) was used as positive control whereas p44/42 ERK was used as negative control. Ctl corresponds to untransfected control cells. A long exposure for PFDN5 is shown on the right. C) Quantification of the biotinylated prefoldin / pVHL expression levels for VHL213wt-, VHL213mut- and VHL172-BirA fusion proteins. Histograms represent the mean ratios of biotinylated PFDN1, PFDN3, PFDN5 and CUL2 proteins (VHL binding partner) on total pVHL expression. For each analyzed protein, the ratio was set as 100% in full-length VHL213wt-expressing cells. Mean±s.d. from three independent experiments, n.s not significant; **, p-value<0.01; ***, p-value<0.001; VHL213 wt vs VHL213 mut and VHL172 for each VHL binding partner, Mann-Whitney test) (PDF) [file pgen.1009183.s003.pdf]

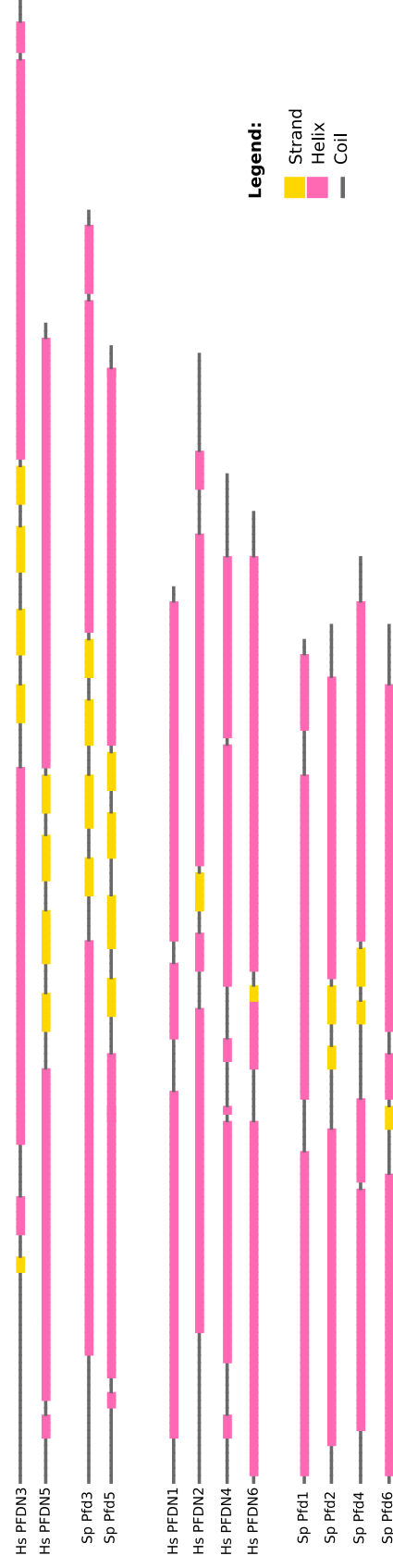

Figure S5

Supplement: S5 Fig — The NH2- and COOH-terminal regions of prefoldin subunits are formed by α-helices (pink) that are connected by β-hairpin linkers. Each β-hairpin linker consists of four short β-strands (yellow) for α prefoldin subunits (PFDN3, PFDN5, Pfd3, Pfd5) and usually one or two short β-strands for β prefoldin subunits (PFDN2, PFDN6, Pfd2, Pfd4, Pfd6). No short β-strands were predicted by PSIPRED between α-helices for prefoldin subunits Hs PFDN1, Hs PFDN4 and Sp Pfd1. Hs: Homo sapiens, Sp: Schizosaccharomyces pombe. (PDF) [file pgen.1009183.s005.pdf]

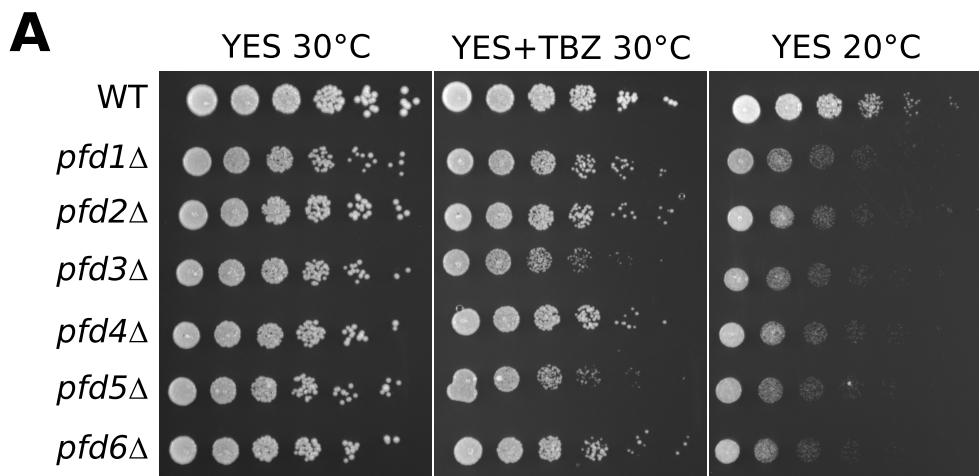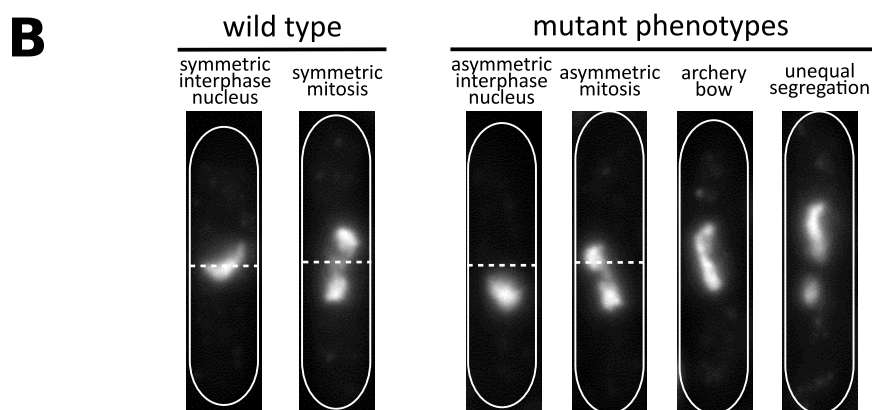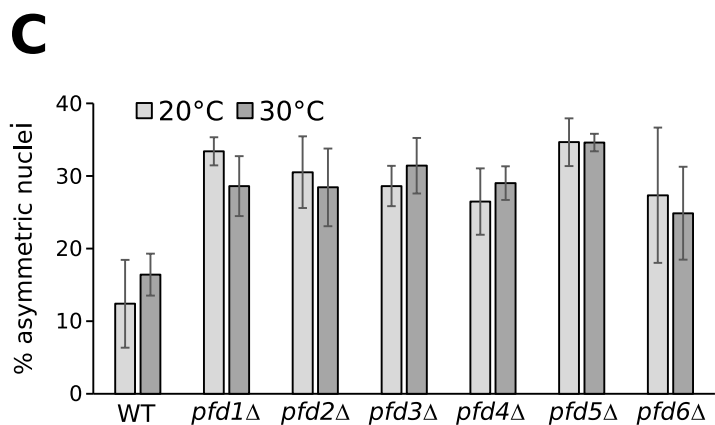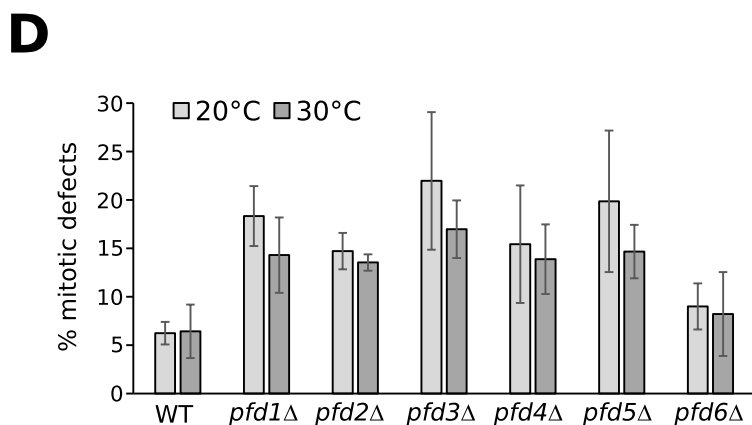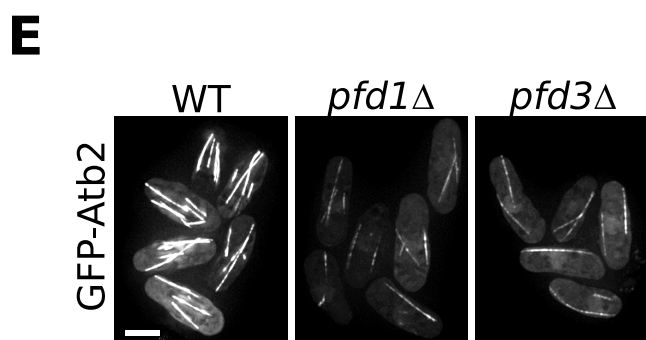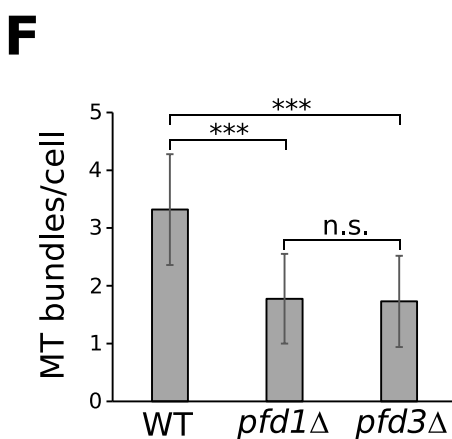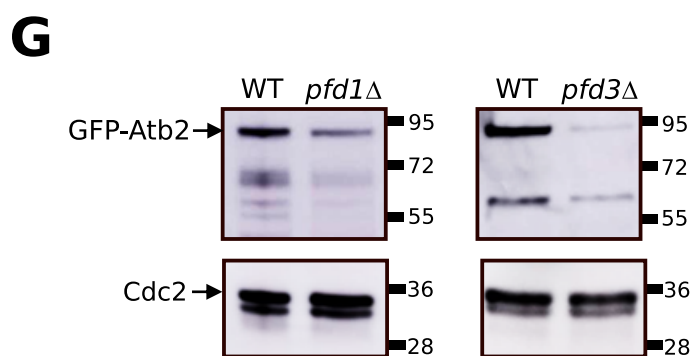

S6 Fig

Supplement: S6 Fig — A) Serial dilutions (1:5) of wild-type and pfd deletion mutants were spotted on YES plates (YES 30°C) or YES plates containing 7.5 mM microtubule-depolymerizing Thiabendazole (YES+TBZ 30°C) at 30°C (2 days) or on YES plates at 20°C for 4 days (YES 20°C). B) Cellular phenotypes of nuclear positions or mitotic defects of fission yeast prefoldin mutants. Percentage of cells showing C) an asymmetric nucleus or D) mitotic defects in WT and prefoldin mutants at 20°C and 30°C (mean±s.d. from three independent experiments). E) Microtubule network organization in WT, pfd1Δ and pfd3Δ cells expressing GFP-tagged α-tubulin (Atb2). Bar: 5 μm. F) Histogram reporting the number of MT bundles observed per cell in WT, pfd1Δ and pfd3Δ strains. (n.s., non significant; ***, p<0.001). G) Western blot analysis of GFP-Atb2 expression in WT, pfd1Δ and pfd3Δ strains. Cdc2 was used as a loading control. (PDF) [file pgen.1009183.s006.pdf]

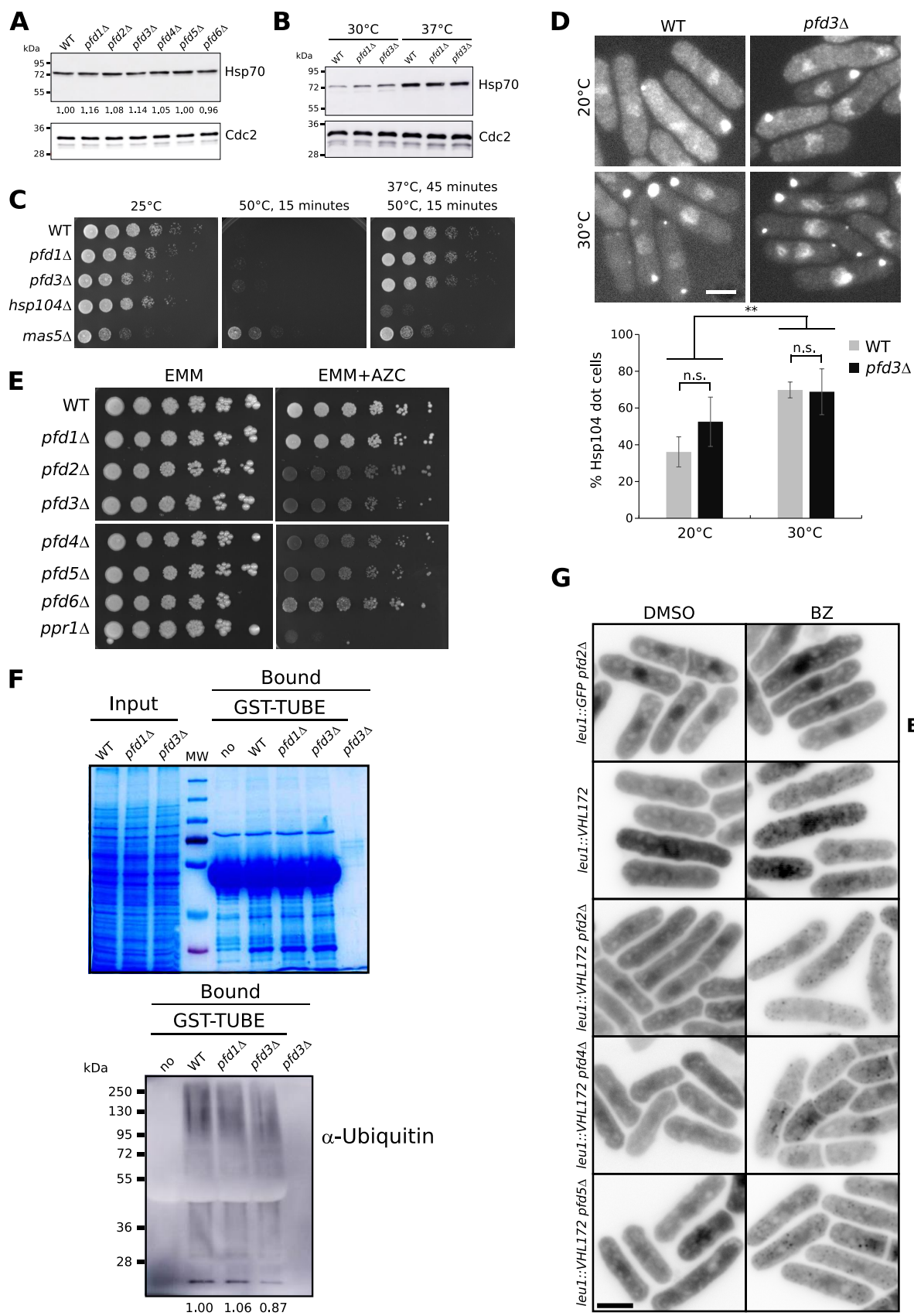

Supplement: S7 Fig — A and B) Steady-state and heat-induced Hsp70 expression levels are similar in wild type and prefoldin mutant strains. A) Western blot analysis of Hsp70 expression levels in wild type (WT) and prefoldin mutants. Cells were grown to exponential growth phase at 30°C to prepare whole cell protein extracts. At the bottom of the Hsp70 gel are indicated the mean relative amounts of Hsp70 in the different strains (WT was set to 1; mean of 3 experiments, no significant difference, Kruskal-Wallis test). B) Western blot analysis of Hsp70 expression levels in wild type (WT) and pfd1Δ and pfd3Δ mutants at 30°C and after a 2-hour heat shock at 37°C. Cells were grown to exponential growth phase at 30°C and cultures were split in two samples. One was left at 30°C (left panel) and the other half was incubated for 2h at 37°C (right panel). Cdc2 was used as a loading control. C) Wild type and prefoldin mutant strains show similar thermotolerance. The growth phenotypes of the wild type (WT) and pfd mutant strains were compared to the thermotolerance-deficient hsp104Δ mutant and to the mas5Δ mutant which exhibits a high Hsf1 constitutive activity. Wild type and mutant cells were grown to mid-log phase and were either spotted on YES plates at 25°C for 3 days (left panel) or shifted to 50°C for 15 minutes before being spotted at 25°C (middle panel). An additional sample was first shifted to 37°C for 45 minutes before being exposed to 50°C (right panel). D) Wild type and pfd3Δ cells exhibit similar Hsp104-GFP distributions. Reversed images (upper panels) and quantification of Hsp104-GFP dot containing cells (bottom panel) of wild-type (WT) or pfd3Δ cells at 20°C or 30°C. E) Wild type and prefoldin mutant strains show similar sensitivity to the proline analog azetidin-2-carboxylic acid (AZC) which promotes general protein misfolding. The growth phenotypes of the indicated wild type (WT) and pfd mutant strains were compared to the AZC-sensitive AZC acetyltransferase deletion mutant ppr1Δ str [file pgen.1009183.s007.pdf]

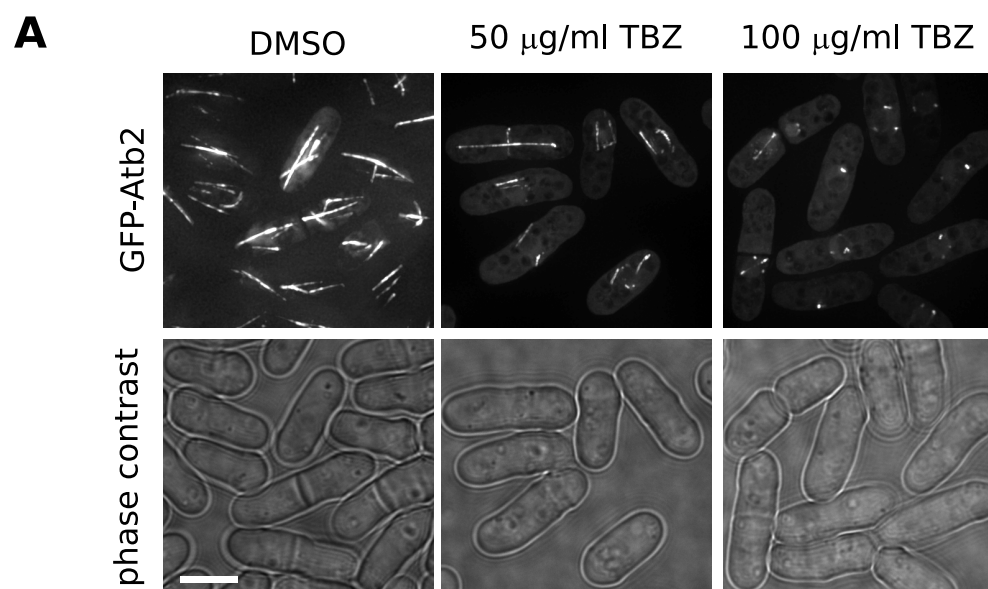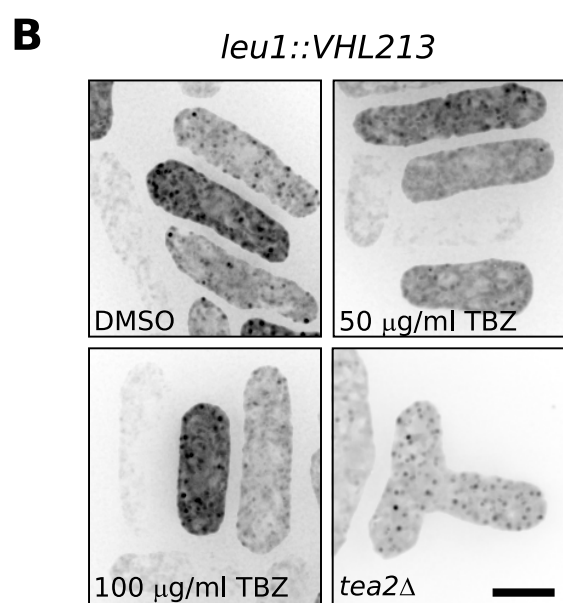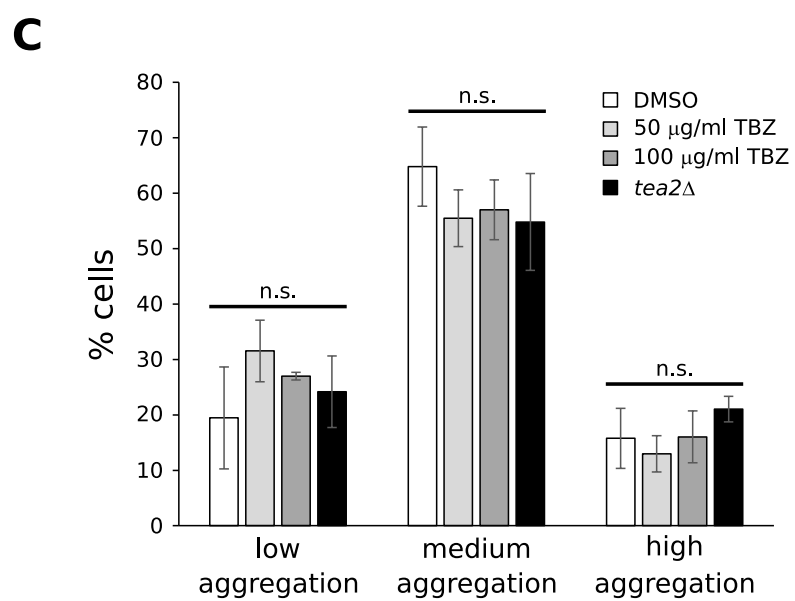

S8 Fig

Supplement: S8 Fig — A) The effect of two concentrations of the MT-depolymerizing drug, thiabendazole (TBZ), on the MT network organization was assayed by imaging GFP-Atb2 (alpha-tubulin) in fission yeast cells: deconvolved GFP fluorescence (upper panels) and phase contrast (lower panels). B and C) The impact of two concentrations of TBZ and of the MT-deficient tea2Δ mutant on pVHL213 aggregation was monitored: B) Reversed deconvolved fluorescent images of GFP-VHL213 expressing cells and C) Histogram representing the percentage of cells with low (<10 aggregates/cell), medium (20–30 aggregates/cells) or high (>30 aggregates/cell) pVHL213 aggregation patterns (mean of 3 independent experiments; DMSO, n = 802; 50 μg/ml TBZ, n = 693; 100 μg/ml TBZ, n = 635; tea2Δ, n = 175). Statistical tests showed no significant difference (n.s.) between the samples. Bars: 5 μm. (PDF) [file pgen.1009183.s008.pdf]

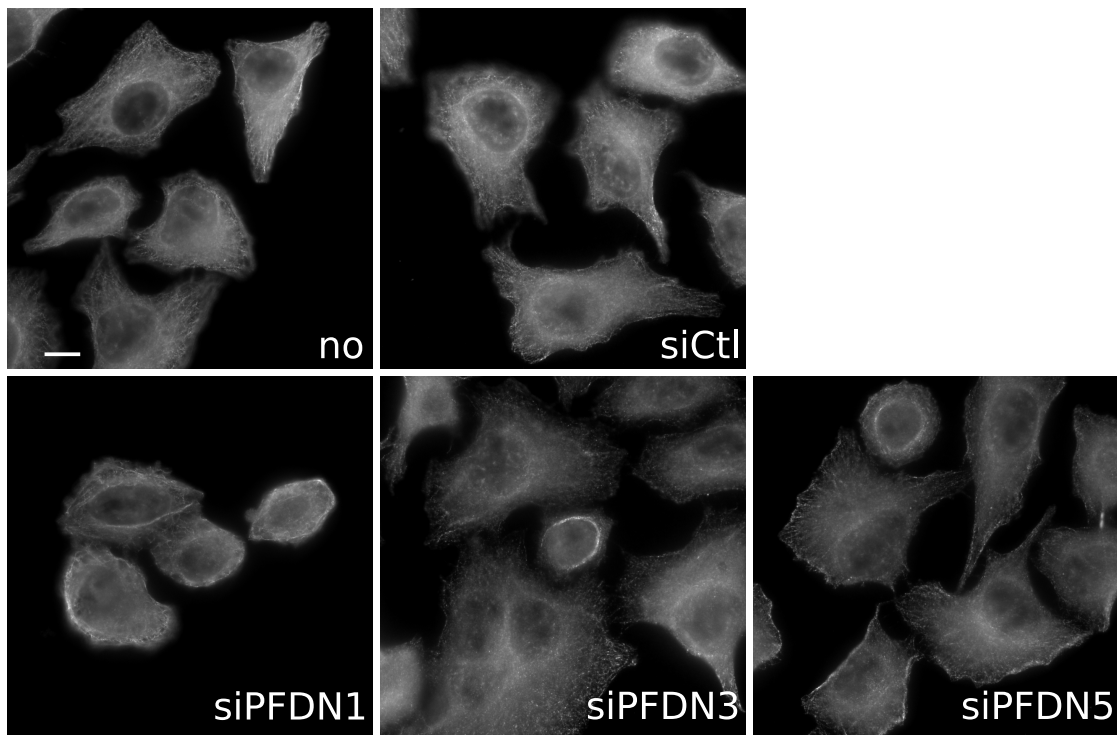

S9 Fig

Supplement: S9 Fig — Representative immunofluorescence images of the microtubule network of untreated (no) HeLa cells or siRNA treated cells with control SiRNA (siCtl) or siRNA targeting either PFDN1, PFDN3 or PFDN5 genes. Bar: 10 μm. (PDF) [file pgen.1009183.s009.pdf]

**A**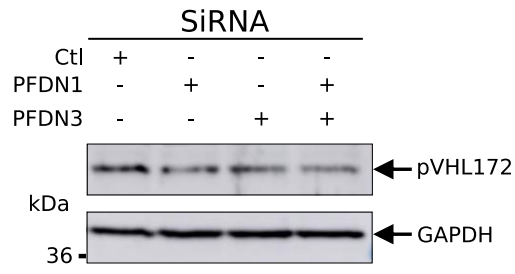**B**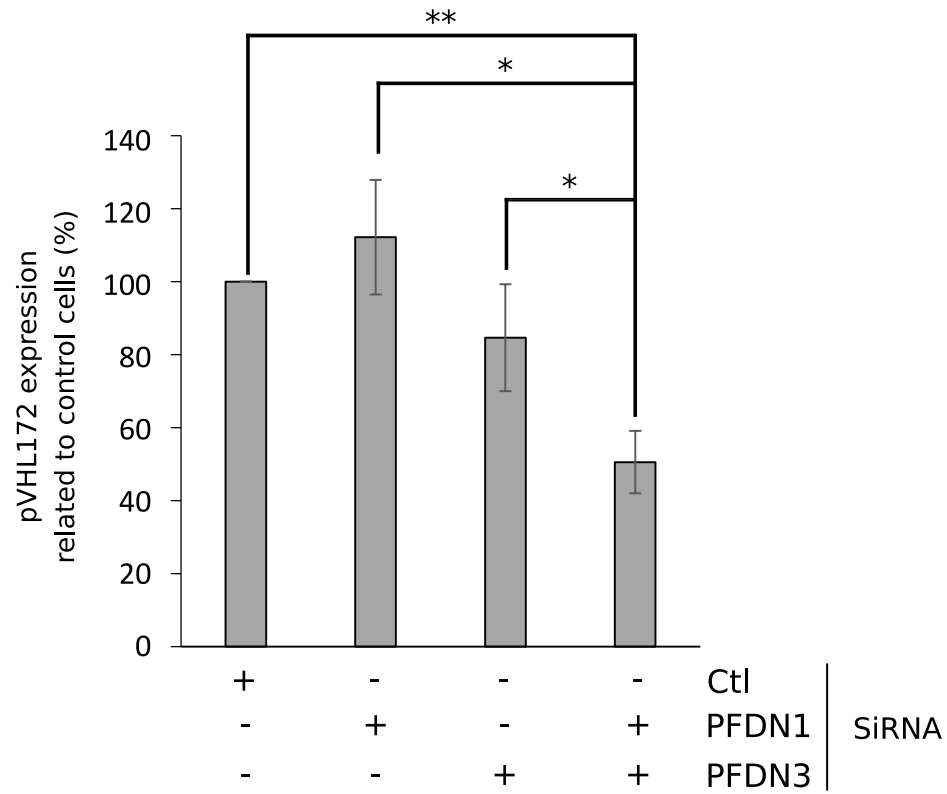

S10 Fig

Supplement: S10 Fig — A) Western blot analysis of pVHL172 expression after siRNA-driven knock-down of PFDN1, PFDN3 or PFDN1+PFDN3 in HeLa cells. B) Histogram representing the pVHL172 levels in PFDN siRNA experiments (mean±s.e.m from at least three independent experiments). “Si Ctl” represents cells transfected with mock siRNA. *, p-value<0.05; **, p-value<0.01. Mann-Whitney test. (PDF) [file pgen.1009183.s010.pdf]

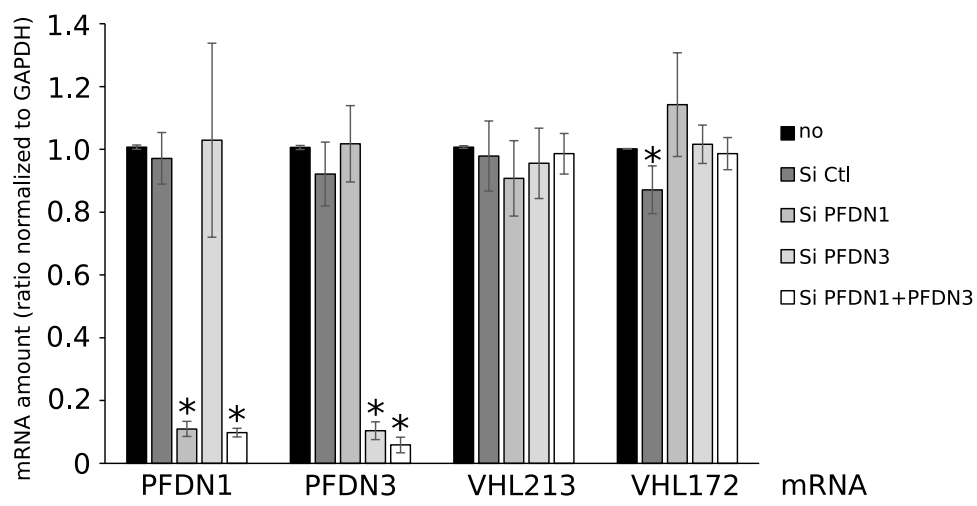

S11 Fig

Supplement: S11 Fig — RT-qPCR analysis of PFDN1, PFDN3, full-length VHL213 and exon-2 deleted VHL172 mRNA expression in HeLa cells treated with PFDN1, PFDN3, PFDN1+PFDN3 siRNA (Si) or control siRNA (Si Ctl) or untreated cells (no; tranfectant only). The GAPDH gene expression was used for normalization (n = 4). The mRNA levels in untreated cells were set to 1 for each condition. No significant differences were observed between the samples except when indicated * (p<0.05); Mann-Whitney test. (PDF) [file pgen.1009183.s011.pdf]

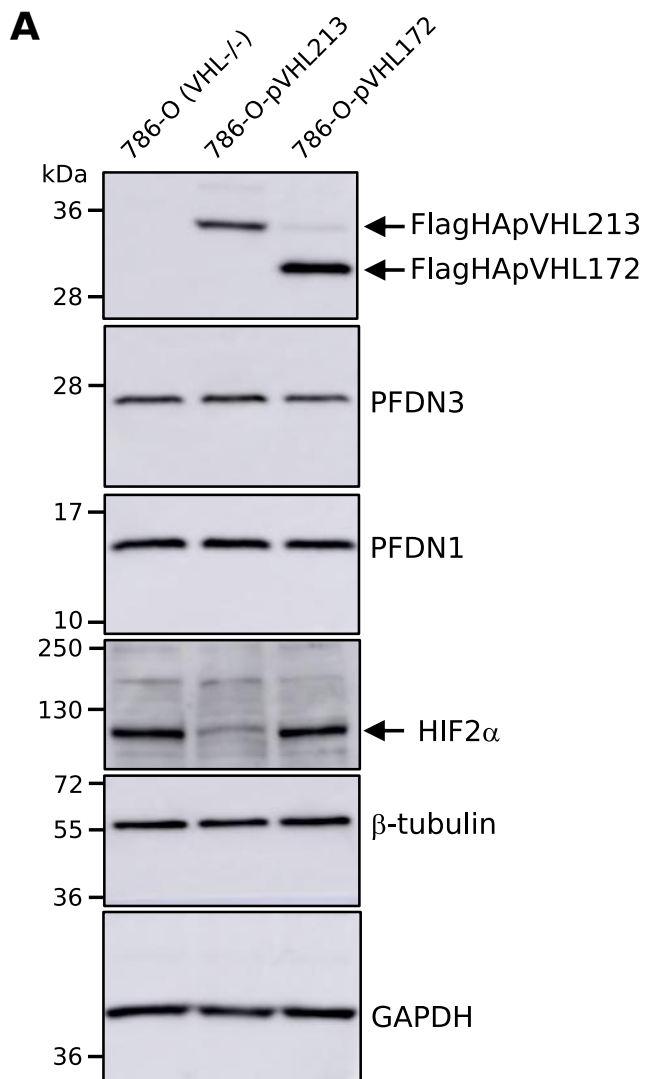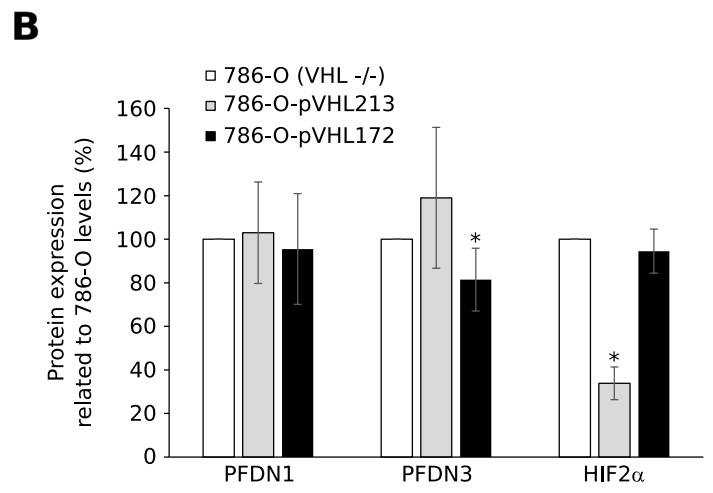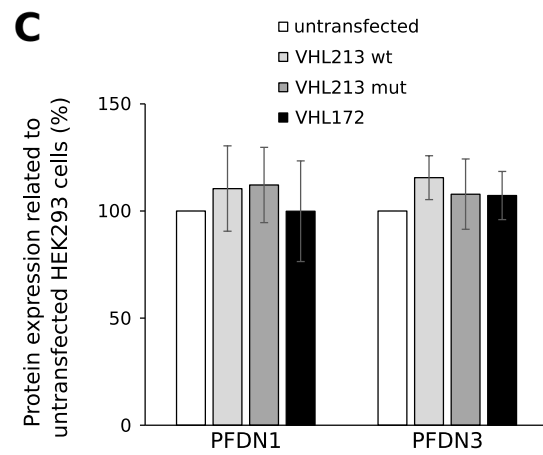

S12 Fig

Supplement: S12 Fig — A) Western blot analysis of PFDN1 and PFDN3 in protein extracts from the 786-O parental cell line (VHL-/-), the 786-O-pVHL213 and 786-O-pVHL172 cell lines [4]. HIF2α, a target of the pVHL213 VBC E3 ligase complex, was used as a positive control for E3 ligase activity whereas GAPDH and β-tubulin were used as loading controls. B) Quantification of the expression levels of PFDN1, PFDN3 and HIF2α. Mean±s.d. from three independent experiments, no significant difference for samples except PFDN3 in 786-O-pVHL172 and HIF2α in 786-O-pVHL213 (*, p-value<0.1; Mann-Whitney test). C) Quantification of PFDN1 and PFDN3 expression levels after a Western blot analysis in total protein extracts from HEK293 cells transiently transfected with wild type VHL213 (VHL213 wt), mutated VHL213 deficient for prefoldin binding (VHL213 mut) or VHL172. The levels of PFDN1 and PFDN3 were set as 100% in the control untransfected cells. No significant difference were observed between all samples (Mann-Whitney test) (PDF) [file pgen.1009183.s012.pdf]

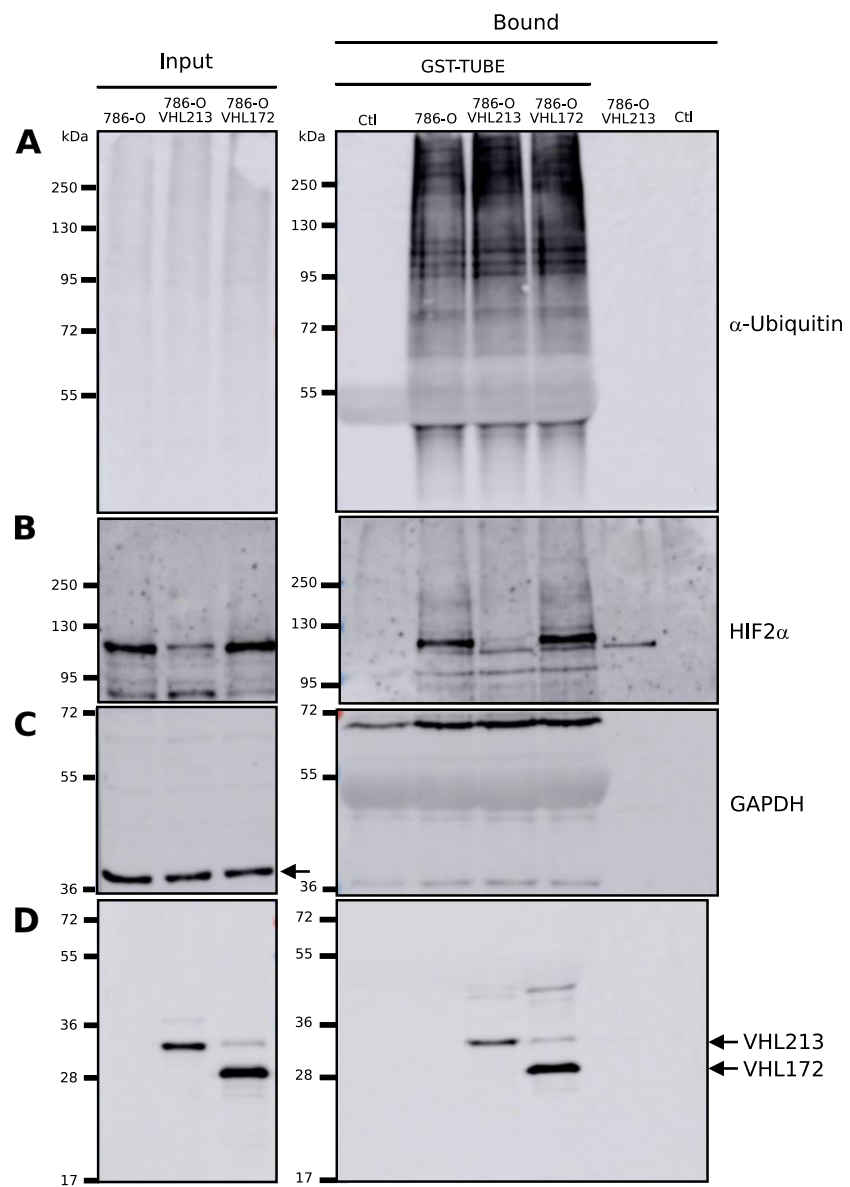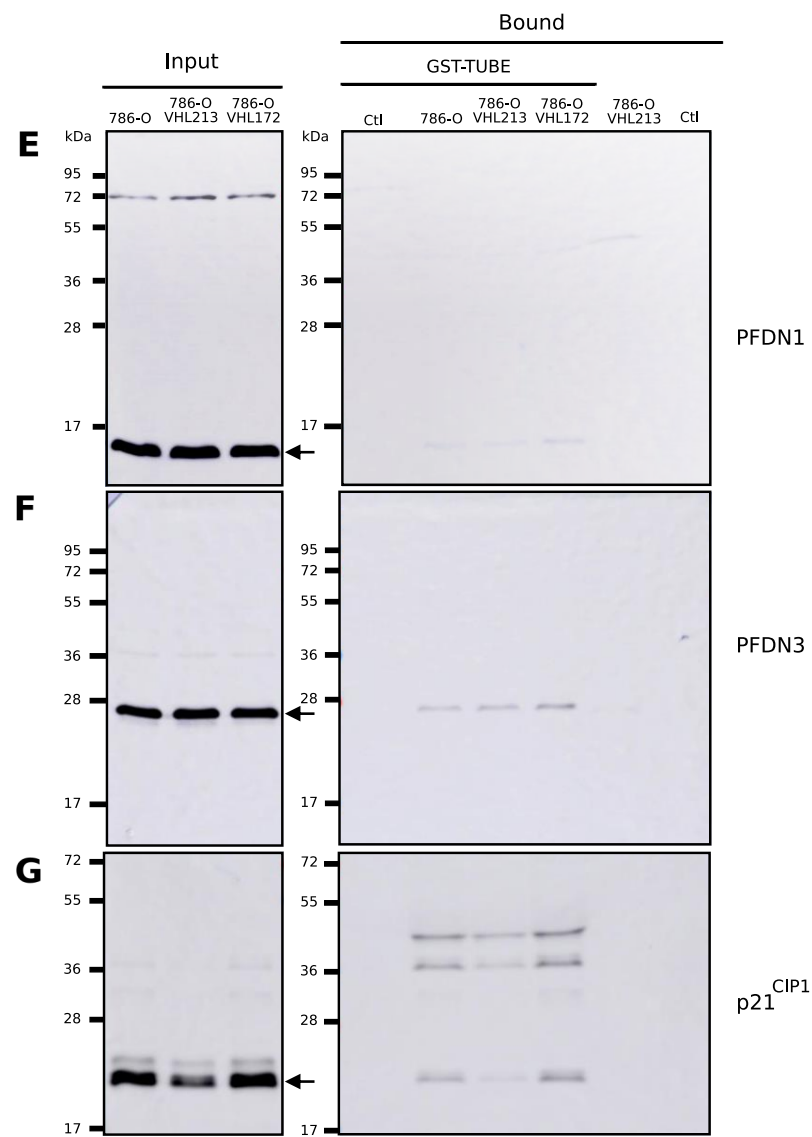

S13 Fig

Supplement: S13 Fig — Western blot analyses of total protein extracts (Input) and of pulled-down (bound) fractions eluted from glutathione-agarose-beads loaded or not with GST-TUBE. The cells lines were 786-O (VHL-/-) and 786-O cells expressing either pVHL213 (786-O VHL213) or pVHL172 (786-O VHL172). As controls (Ctl) in pulled-down fractions, no protein extract was loaded on a GST-TUBE-loaded beads (first lane) or only on unloaded glutathione-agarose beads (last lane). Another negative control was a 786-O VHL213 extract incubated with unloaded gluthathione-agarose beads (penultimate lane). The following proteins were detected in Western blots using specific antibodies: A) total ubiquitinated proteins (using P4D1 antibody), B) HIF2α, C) GAPDH (negative control), D) pVHL isoforms, E) PFDN1, F) PFDN3, G) p21CIP1. (PDF) [file pgen.1009183.s013.pdf]
