# Supplementary material for: The prefoldin complex stabilizes the von Hippel-Lindau protein against aggregation and degradation
Source: PLoS Genet. 2020 Nov 2;16(11):e1009183. doi: 10.1371/journal.pgen.1009183 (PMC7660911; doi:10.1371/journal.pgen.1009183)
Supplement: S4 Fig — A) Venn diagram comparison of proteins identified by LC-MS/MS in GFP (control) and VHL-GFP affinity-purification extracts. B) Most significant over-represented functional categories classed by gene ontology (GO) for VHL-specific interactors. (PDF) [file pgen.1009183.s004.pdf]

A

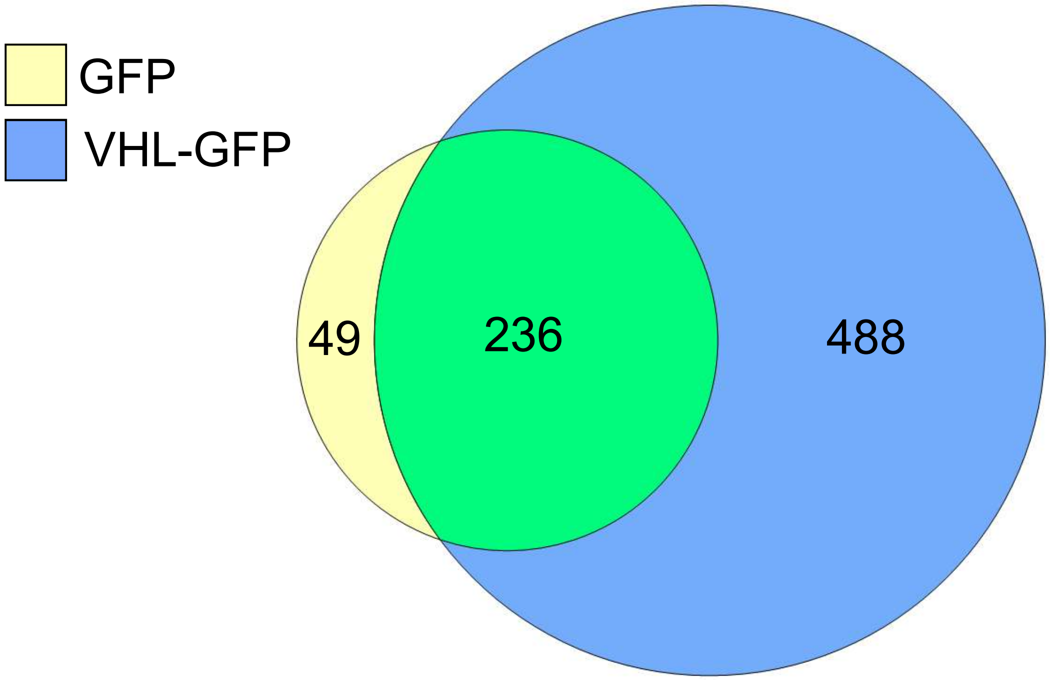

B

Category	GO ID	GO term	*P*-value
Biological process	0006457	Protein folding	7.2E-06
Cellular component	0005829	Cytosol	6.3E-21
Molecular function	0005524	ATP binding	1.9E-10
