# Supplementary material for: The prefoldin complex stabilizes the von Hippel-Lindau protein against aggregation and degradation
Source: PLoS Genet. 2020 Nov 2;16(11):e1009183. doi: 10.1371/journal.pgen.1009183 (PMC7660911; doi:10.1371/journal.pgen.1009183)
Supplement: S2 Table — Schizosaccharomyces pombe prefoldin subunit encoding genes (pfd1 to pfd6) were named according to their human counterparts (Human homologue). The amino acid identities with prefoldin subunits of different model organisms (budding yeast Saccharomyces cerevisiae, the nematode Caenorhabditis elegans, the fruit fly Drosophila melanogaster, the mouse Mus musculus and human Homo sapiens) are indicated on the right. (PDF) [file pgen.1009183.s015.pdf]

S2 Table: Sequence conservation of prefoldin subunits in model eukaryotes.

Gene name	gene ID	alias	subunit type	product size (aa)	Human homologue	% amino acid identity				
						Homo sapiens	*Mus musculus*	*Drosophila melanogaster*	*Caenorhabditis elegans*	*Saccharomyces cerevisiae*
pfd1	SPBC1D7.01	none	beta	112	*PFDN1*	21.4	21.4	19.6	25.9	28.4
pfd2	SPAC227.10	none	beta	114	*PFDN2*	31.6	31.6	29.0	21.1	41.4
pfd3	SPAC3H8.07c	*pac10*	alpha	169	*PFDN3*	43.2	43.2	39.3	34.3	44.9
pfd4	SPAC227.05	none	beta	123	*PFDN4*	34.2	32.5	26.7	34.7	33.6
pfd5	SPBC215.02	*bob1*	alpha	154	*PFDN5*	45.2	45.2	32.9	32.2	35.8
pfd6	SPAC3A11.13	none	beta	114	*PFDN6*	36.8	36.8	32.5	29.5	42.1

Table S2
