# Supplementary material for: The prefoldin complex stabilizes the von Hippel-Lindau protein against aggregation and degradation
Source: PLoS Genet. 2020 Nov 2;16(11):e1009183. doi: 10.1371/journal.pgen.1009183 (PMC7660911; doi:10.1371/journal.pgen.1009183)
Supplement: S3 Table — (DOCX) [file pgen.1009183.s016.docx]

**S3 Table: *S. pombe* strains used in this study.**

| **Number** | **Genotype** | **Reference** |
| --- | --- | --- |
| **XLG029** | *h- leu1-32* | Lab stock |
| **XLG106** | *h- tea2::his3^+^ his3-D1 ade6- ura4-D18 leu1-32* | Kind gift of P. Nurse |
| **XLG218** | *h- nmt1-GFP-Atb2.kanR* | Kind gift of I. Hagan |
| **XLG841** | *h- leu1-32 ade6- ura4-D18 hsp104-GFP-S65T:kan* | Kind gift of P. Sunnerhagen |
| **XLG904** | *h- leu1::[nmt1-GFP-VHL213.ura4^+^] ura4-D18* | This study |
| **XLG939** | *h+ pac10/pfd3(SPAC3H8.07c )::kanMX6 ura4-D18 ade6-M210 leu1-32* | Kind gift of R. Hartmann-Petersen |
| **XLG941** | *h+ pfd6(SPAC3A11.13)::kanMX6 ura4-D18 ade6-M210 leu1-32* | Kind gift of R. Hartmann-Petersen |
| **XLG943** | *h+ bob1/pfd5(SPBC215.02)::kanMX6 ura4-D18 ade6-M210 leu1-32* | Kind gift of R. Hartmann-Petersen |
| **XLG944** | *h+ pfd4(SPAC227.05)::kanMX6 ura4-D18 ade6-M210 leu1-32* | Kind gift of R. Hartmann-Petersen |
| **XLG1024** | *h+ nas6::kanMX6 leu1-32 ade6-M210 ura4-D18* | Kind gift of R. Hartmann-Petersen |
| **XLG954** | *h+ mas5::ura4^+^ ade6-210 ura4-D18 leu1-32* | Kind gift of S. Oliferenko |
| **XLG957** | *h+ hsp104::kanMX6 ade6-210 ura4-D18 leu1-32* | Kind gift of D. Coudreuse |
| **XLG958** | *h+ pfd2(SPAC227.10)::kanMX6 ade6-210 ura4-D18 leu1-32* | Kind gift of D. Coudreuse |
| **XLG1018** | *h- leu1::[nmt1-GFP-VHL172.ura4^+^] ura4-D18* | This study |
| **XLG1039** | *h- pfd4::kanMX6 leu1::[nmt1-GFP-VHL172.ura4^+^] ura4-D18* | This study |
| **XLG1040** | *h- pfd5::kanMX6 leu1::[nmt1-GFP-VHL172.ura4^+^] ura4-D18* | This study |
| **XLG1043** | *h- pfd4::kanMX6 leu1::[nmt1-GFP-VHL213.ura4^+^] ura4-D18* | This study |
| **XLG1045** | *h- pfd6::kanMX6 leu1::[nmt1-GFP-VHL213.ura4^+^] ura4-D18* | This study |
| **XLG1048** | *h- pfd5::kanMX6 leu1::[nmt1-GFP-VHL213.ura4^+^] ura4-D18* | This study |
| **XLG1053** | *h- nas6::kanMX6 leu1::[nmt1-GFP-VHL213.ura4^+^] ade6- ura4-D18* | This study |
| **XLG1058** | *h- pfd3::kanMX6 leu1::[nmt1-GFP-VHL213.ura4^+^] ura4-D18* | This study |
| **XLG1066** | *h- pfd2::kanMX6 leu1::[nmt1-GFP.ura4^+^] ura4-D18* | This study |
| **XLG1067** | *h- pfd2::kanMX6 leu1::[nmt1-GFP-VHL172.ura4^+^] ura4-D18* | This study |
| **XLG1068** | *h- pfd2::kanMX6 leu1::[nmt1-GFP-VHL213.ura4^+^] ura4-D18* | This study |
| **XLG1075** | *h- pfd1(SPBC1D7.01)::kanMX6 ura4-D18 leu1-32* | This study |
| **XLG1079** | *h- leu1::[nmt1-GFP.ura4^+^] ura4-D18* | This study |
| **XLG1080** | *h- nas6::kanMX6 leu1::[nmt1-GFP.ura4^+^] ade6- ura4-D18* | This study |
| **XLG1081** | *h- pfd1::kanMX6 leu1::[nmt1-GFP-VHL213.ura4^+^] ura4-D18* | This study |
| **XLG1087** | *h- pfd3::kanMX6 nmt1-GFP-Atb2.kanR* | This study |
| **XLG1089** | *h- pfd1::kanMX6 nmt1-GFP-Atb2.kanR* | This study |
| **XLG1095** | *h- pfd3::kanMX6 Hsp104-GFP.kanMX6 ura4-D18 leu1-32* | This study |
| **XLG1111** | *ARC-Dppr1 (ppr1::ura4^+^)* | Kind gift of T. Hiroshi |
